# Innovative Approach to Sustainable Marine Invertebrate Chemistry and a Scale-Up Technology for Open Marine Ecosystems

**DOI:** 10.3390/md16050152

**Published:** 2018-05-06

**Authors:** Pinelopi Vlachou, Géraldine Le Goff, Carolina Alonso, Pedro A. Álvarez, Jean-François Gallard, Nikolas Fokialakis, Jamal Ouazzani

**Affiliations:** 1 Department of Pharmacognosy & Natural Products Chemistry, Faculty of Pharmacy, National and Kapodistrian University of Athens, 15771 Athens, Greece; pvlachou@pharm.uoa.gr (P.V.); fokialakis@pharm.uoa.gr (N.F.); 2 Institut de Chimie des Substances Naturelles ICSN, Centre National de la Recherche Scientifique, Avenue de la Terrasse, 91198 Gif-sur-Yvette, France; geraldine.legoff@cnrs.fr (G.L.G); Jean-francois.Gallard@cnrs.fr (J.-F.G.); 3 iMARE natural S.L. Avda. Habana, 10, 18600 Motril, Spain; carolina.imare@gmail.com (C.A.); pedro.imare@gmail.com (P.A.Á.)

**Keywords:** *Crambe crambe*, solid-phase extraction, guanidine alkaloids, crambescidins and crambescins, Somartex

## Abstract

Isolation of marine compounds from living invertebrates represents a major challenge for sustainable and environmentally friendly exploitation of marine bio-resources. To develop innovative technology to trap invertebrate compounds in the open sea, the proof of concept of a system combining external continuous circulation of water with XAD-amberlite solid-phase extraction was validated in an aquarium. In this work, we reported the elicitation of guanidine alkaloid production of *Crambe crambe* in the presence of *Anemonia sulcata*, both collected from the Mediterranean Sea. Besides the previously reported crambescidin 359 (**1**), and crambescidin acid (**2**), three new compounds were isolated; one carboxylated analog of **1** named crambescidin 401 (**3**), and two analogs of crambescin B, crambescin B 281 (**4**) and crambescin B 253 (**5**). Based on these results, a technology named Somartex^®^ for “Self Operating MARine Trapping Extractor” was patented and built to transfer the concept from closed aquarium systems to open marine ecosystems.

## 1. Introduction

The marine environment represents a diverse and abundant reservoir of valuable molecules. Oceans cover more than 70% of the planet, extending to depths of more than 3 km and host an exceptional biodiversity consisting of more than 90% of animal phyla as well as a rich biodiversity of plants, algae and microorganisms. Technical diving, remote control vehicles and deep-sea exploration vehicles remaining of limited use due to their cost, autonomy and complexity. As a result, major discoveries have been concentrated around the shallow waters, while the greater depths of the ocean remain far less explored and understood.

During the recent decades, not only terrestrial biodiversity but also marine species, especially marine invertebrates and microorganisms, have been investigated for their potential pharmaceutical applications. However, compared to their terrestrial counterparts, few marine-derived drugs have progressed through to the later stages of clinical trials [1].

The major limiting factor in marine invertebrate chemistry is the difficulty to access the bioresource. In addition to the difficulties involved in harvesting the invertebrates, these sessile animals face various environmental and anthropogenic threats. Many are endangered species and protected through national, regional and international conventions. Attempts to cultivate marine invertebrates or their cells have not, to date, provided a satisfactory solution to the problem of sourcing [2,3,4]. This is due not only to the low yield and slow growth rate but also a result of taking the invertebrates out of their natural environment, which implies removing natural physico-chemical constraints and the presence of symbionts, resulting in the cultivated invertebrates not being able to produce the desired target compounds.

The future challenge is to ensure the protection of marine invertebrate biodiversity while meeting the need to provide the drug pipeline with marine natural scaffolds and bioactive compounds.

The trapping of invertebrate molecules in their natural habitat, without harvesting the organism, represents a novel approach and a potential solution to the problem at hand to which no efforts have yet been dedicated.

In this paper, we report a proof of concept for an innovative strategy enabling the trapping of molecules from invertebrates maintained alive in an aquarium. This approach allows the isolation and the structural elucidation of the produced compounds without applying any stress or injury either to the animal or to its environment. To validate the approach in open marine ecosystems, a specific technology, named Somartex^®^ (Self Operating MARine Trapping Extractor, Maximator SAS, Rantigny, France) was patented and built for testing in different marine locations and depths.

## 2. Results

### 2.1. From the Observation to the Proof of Concept

Our strategy is based on the observation that when the red encrusting sponge *Crambe crambe* from the Mediterranean Sea was introduced into an aquarium containing the cnidarian *Anemonia sulcata*, the anemonia died within 48 h, whereas the *Crambe crambe* sponge seemed not to be affected. One possible explanation is that *Crambe crambe* produces compounds in these conditions that are toxic to anemonia.

To verify this hypothesis, the trapping technology was applied, in similar conditions, to four aquariums. The first aquarium (Aq-1) did not contain any invertebrate and served as a control. The second aquarium (Aq-2) contained 20 *Anemonia sulcata*, the third aquarium (Aq-3) contained stones holding *Crambe crambe* sponge samples, the fourth (Aq-4) contained 20 *Anemonia sulcata* to which stones holding *Crambe crambe* sponge were added.

**Figure 1:**
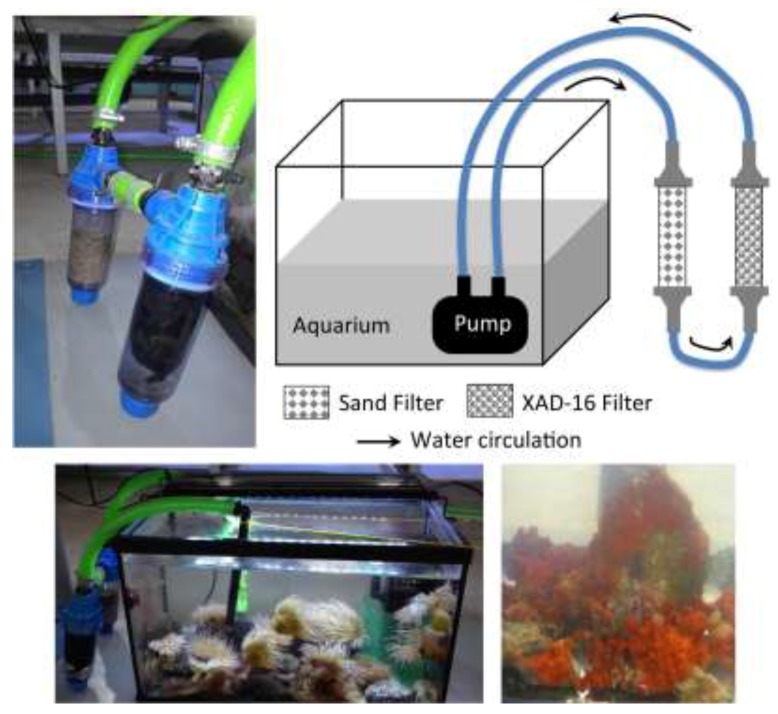
Design and pictures of the experimental assembly.

In each aquarium, water was circulated continuously in a filter containing Amberlite XAD-16 resin polymeric absorbent (Figure 1).

After 48 h, the Amberlite XAD-16 (≈50 g) is recovered from the filters and extensively washed with distilled water. According to the recovered quantities and analytical profiles, only the MeOH extract of Aq-4 offered analytical profiles and yielded quantities sufficient for advanced isolation and structural elucidation tasks (160 mg). In general, we applied the in situ XAD-16 solid-phase extraction to microbial secondary metabolites. Among the many advantages of this method, it allows the isolation of the metabolic intermediates and the proposal of biosynthetic pathways [5,6].

**Figure 2:**
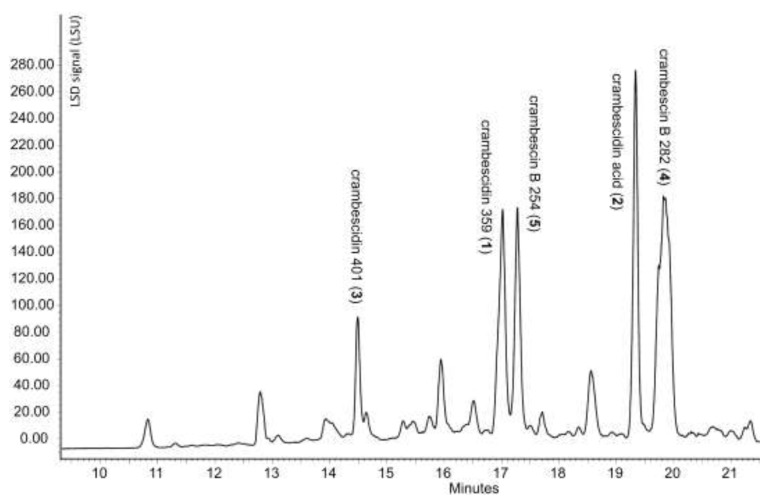
HPLC analysis of the MeOH extract of aquarium 4 (Aq-4) under LSD detection.

As shown in Figure 2, the HPLC-ELSD chromatogram of the MeOH extract shows various peaks, which were isolated by a combination of flash chromatography and semi-preparative HPLC (see materials and methods section).

### 2.2. Isolation and Structural Elucidation

**Figure 3:**
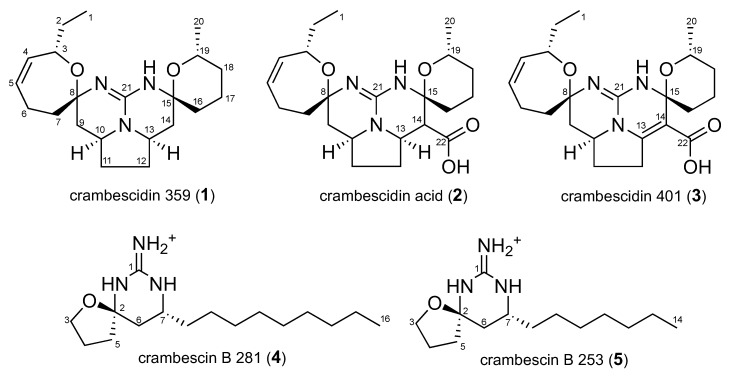
Structures of the isolated compounds **1** to **5**.

Chromatographic separation of major compounds present in the extract (Figure 2) led to the isolation of five compounds in mg scale. Their structure elucidation was performed based on 1D, 2D NMR and high-resolution mass spectrometry experiments (Figure 3).

Molecular formula C_21_H_33_N_3_O_2_ of **1** was established based on HR-ESI-MS (*m/z* 360.2651 [M+H]^+^). This compound was identified as crambescidin 359 based on the identical NMR data (Supplementary Material, Table S30) to those previously reported by Breakman et al. [7].

Compound **2** had a HR-ESI-MS of *m/z* 404.2549 [M+H]^+^ corresponding to the molecular formula C_22_H_33_N_3_O_4_. The ^1^H and ^13^C NMR spectra (Table 1) are identical to those reported for crambescidin acid, previously reported by McMahon et al. [8]. Crambescidin acid (**2**) is herein reported for the first time to be isolated from *Crambe crambe* but has also been reported by Overman et al. as the key intermediate in the total synthesis of crambescidins 359 and 431 [9]. The authors demonstrated that the zwitterionic guanidinium carboxylate at **2** readily decarboxylates to form crambescidin 359 (**1**), more easily than under basic conditions.

**Figure 4:**
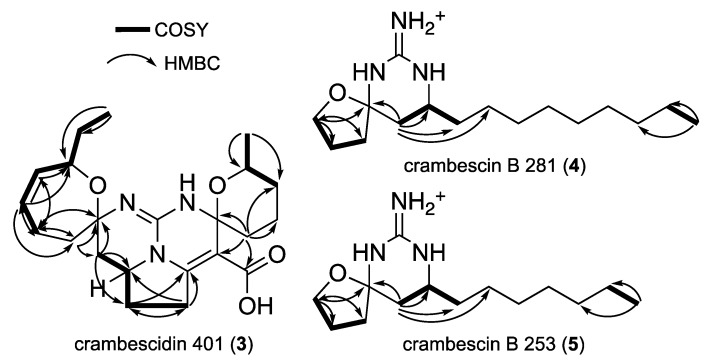
COSY and key HMBC correlations for compounds **3** to **5**.

The new crambescidin derivative **3**, named crambescidin 401 had a molecular formula C_22_H_31_N_3_O_4_ as deduced from HR-ESI-MS (*m/z* 402.2393 [M+H]^+^). Its ^1^H and ^13^C NMR spectra were very similar to those of **2**. The main difference was the absence of ^1^H NMR signals corresponding to H-13 and H-14 in **3**. This observation, together with the comparison of molecular formulae, suggested the presence of a double bond in C-13/C-14 position. This was supported by the chemical shifts of C-13 and C-14 δ_C_ 160.7 and δ_C_ 123.6 respectively. as well as by HMBC correlations (Figure 4) from H-12 (δ_H_ 3.38) to C-13 and from H-16 (δ_H_ 3.01-3.08) to C-14 (Table 1).

**Figure 5:**
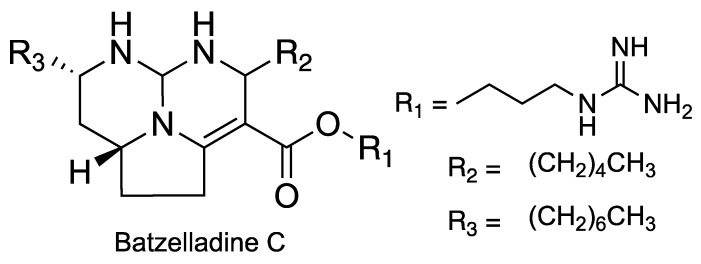
Structures of batzelladine C.

Compound **3** is, to our knowledge, the first representative of the pentacyclic guanidinium alkaloids with a double bond between C-13 and C-14. Only the tricyclic batzelladine C, isolated from Poecilosclerida sponges, bears the double bond at the same position (Figure 5) [10].

The molecular formula C_16_H_31_N_3_O of **4** was determined by HR-ESI-MS (*m/z* 282.2545 [M+H]^+^). The NMR data are listed in Table 2. The molecular formula accounts for three degrees of unsaturation, corresponding to a spiroaminal moiety (C-2, δ_C_ 88.0 ppm) linked to a cyclic guanidine moiety (C-1, δ_C_ 154.4 ppm). The ^13^C NMR revealed the presence of 16 carbons which, in combination with HSQC spectrum, can be categorized as one methyl carbon (δ_C_ 14.2), eleven sp3 methylene groups (including one oxygen-bearing methylene (δ_C_ 67.3), one nitrogen-bearing methine (δ_C_ 47.1), one spiro (δ_C_ 88.0) and one guanidine (δ_C_ 154.4). HMBC correlations are listed in Table 2.

The COSY correlations from H-3 to H-5 allowed building the tetrahydrofuran moiety (Figure 4). HMBC correlations from H-3 and H-6 to C-2 showed the connection of the spiroaminal moiety with the cyclic guanidine through the spiro carbon C-2 (δ_C_ 88.0). The aliphatic from C-8 to C-16 was then connected to the cyclic guanidine, based on COSY correlations from H-6 to H-8 and by HMBC correlations from H-6 to C-6, C-7 and C-9.

Molecular formula C_14_H_27_N_3_O of **5**, a new analog of **4**, was determined by HR-ESI-MS (*m/z* 254.2232 [M+H]^+^). The main difference between the two compounds is the length of the carbon chain holding 7 carbons in **5** and 9 in **4**. The ^1^H, ^13^C NMR data and the HBMC correlations of **5** are shown in Table 2.

**Scheme 1:**
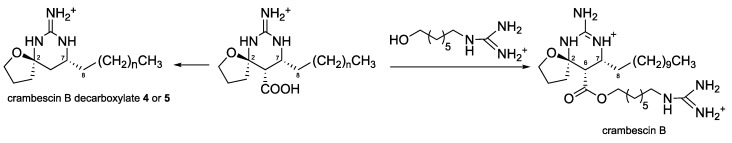
Structure of crambescin B and hypothetic biosynthesis of **4** or **5**.

Compounds **4** and **5** have never been previously described and are related to crambescin B which was isolated in 1990 by Breakman et al. from the sponge *Crambe crambe* [11]. In crambescin B, a guanidino-alkyl ester attached to C-6 indicating that **4** and **5** should be considered as originated from a common biosynthetic precursor (Scheme 1).

Crambescin B derivatives were synthetized by Nishikawa et al. in 2016 [12]. Among them, the crambescin decarboxylate is similar to **4** and **5** but the alkyl chain at C-7 consisted of 11 carbons. The authors highlighted that the relative stereochemistry of crambescin B decarboxylate could not be determined by NOESY analysis, which was confirmed by our data. However, they assumed that the stereochemistry at the C-2 spiro center would be *R* based on the reaction mechanism of the key synthetic steps. Nishikawa et al. [12] synthetized the (+) and (-)-crambescin B decarboxylate. The *α*^25^_D_ + 103.8 (*c* 0.1, CHCl_3_) for **4** and + 124.2 (*c* 0.05, CHCl_3_) for **5**, combined with the supposed *R* configuration of the spiro center C-2 indicate that the absolute configuration of C-2 and C-7 in **4** and **5** is probably *2R-7R*. Attempts to record ECD spectra for **4** and **5** failed to offer exploitable results confirming the difficulty to establish the absolute configuration of crambescin B derivatives since their discovery in 1990.

### 2.3. Building Innovative Equipement for Open Sea Investigation

**Figure 6:**
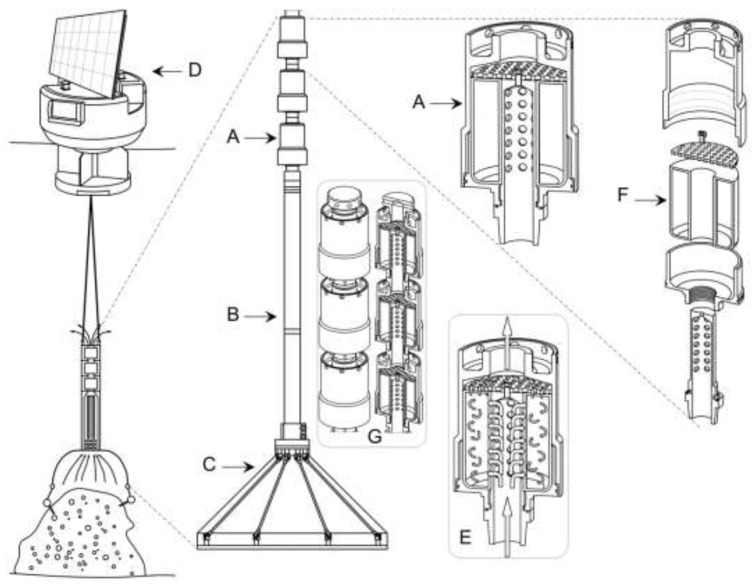
Drawing of Somartex^®^. (A) trapping module, (B) submersible pump, (C) cover dome, (D) Somartex platform, (E) water circulation inside the module. The water is forced to pass through the trapping material (ex. XAD resin) present in the (F) compartment, through the holed tube. The water can then reach the second module through the holed grid, (G) superposition of different modules that may contain different means of molecular trapping.

Beyond the isolated compounds and the three new natural products identified, the design of this experiment offers a proof of concept that molecules could be isolated from marine invertebrates without harming the bio-resources. The invertebrate brought from the sea and maintained in an aquarium for the duration of the experiment could be returned to its original habitat. However, the challenge, that represents the greatest interest in terms of environmental sustainability, is to transpose such a process to the open sea. The invertebrates will be kept in their natural habitat, together with their natural symbionts, and the extraction device will be brought to them with a minimal injury to the animal and minimal harm to the environment. This is the idea behind the development, patenting and construction of Somartex^®^ [13], which will soon be operational and will be tested in diverse locations (Figure 6).

## 3. Discussion

This study shows that the production of secondary metabolites by *Crambe crambe* is probably elicited by the presence of *Anemonia sulcata*, leading to intoxication by crambescidin and crambescin derivatives followed by a rapid death of all anemonia present. None of the compounds reported herein were detected in aquariums containing separately the two invertebrates, and no injury was observed. These experiments prove that secondary metabolites are secreted by the invertebrate to the surrounding water and could be captured by trapping materials, such as XAD resins. This represents the fundamental principle and the proof of concept for the development of Somartex^®^ technology.

The key step in the design of our aquarium experiment is the external continuous circulation of water. This strategy, often used for microbial cultivation, involves a gradual depletion of the compounds from the medium, leading to the trapping of early metabolic intermediates, which unlock the metabolic repression and increased the recovery yield of the resin. In their efforts to investigate sponge-bacteria interactions (*Aplysilla rosea* challenged by its associated bacterium *Streptomyces* ACT-52A), Zhang et al. has used XAD7 packed in cheesecloth bags and introduced in an aquarium that contained the sponge and to which a microorganism was added [14]. Unfortunately, in this batch experiment, no compounds were isolated in sufficient quantity to be fully identified.

The continuous circulation, also applied in Somartex^®^, has another advantage. The dome covering the sampling area can be extended from 40 cm diameter and 40 cm height to 124 cm diameter and 100 cm height. This allows the selection of a unique specimen or a colony of invertebrates. The molecules collected can either come from a single invertebrate or from several species, each containing its own symbionts. This will provide information about the intra- and intermolecular communication network. This will hopefully mark the start of a new era in the growing field of chemical ecology, as well as promoting the sustainable valorization of marine compounds.

## 4. Materials and Methods

### 4.1. General Experimental Procedures

Optical rotations [α]_D_ were measured using an Anton Paar MCP-300 polarimeter at 589 nm (Anton Paar, Les Ulis, France). The IR spectra were obtained using a Perkin-Elmer Spectrum 100 model instrument (Perkin-Elmer, Villebon-sur-Yvette, France). NMR experiments were performed using a Bruker Avance III 600 MHz spectrometer equipped with a TCi cryo-probe head for compounds **2** and **3**, and a Bruker Avance 500 MHz spectrometer for compounds **1**, **4** and **5** (Bruker, Vienna, Austria). All the spectra were acquired in CD_3_OD (δ_H_ 3.31 ppm and δ_C_ 49.15 ppm) at 300 K. High-resolution mass spectra were obtained on a Waters LCT Premier XE spectrometer equipped with an ESI-TOF (electrospray-time of flight) by direct infusion of the purified compounds (Waters SAS, Saint-Quentin-en-Yvelines, France). Pre-packed silica gel Redisep columns were used for flash chromatography using a Combiflash-Companion chromatogram (Serlabo, Entraigues-sur-la-Sorgue, France). All other chemicals and solvents were purchased from SDS (SdS, Peypen, France).

Analytical HPLC system consisted of an Alliance Waters 2695 controller coupled with a PhotoDiode Array Waters 2996, an evaporative light-scattering detector ELSD Waters 2424 detector and a mass detector Waters QDa. Sunfire C_18_ column (4.6 × 150 mm, 3.5 μm) was used with a flow rate of 0.7 mL/min. The elution gradient consisted of a linear gradient from 100 % solvent A to 100% solvent B in 40 min, then 10 min at 100% B (Solvent A: H_2_O + 0,1 HCOOH, Solvent B: ACN + 0,1% HCOOH). Preparative HPLC was performed on a semi-preparative Sunfire C_18_ column (10 × 250 mm, 5 μm) using a Waters autosampler 717, a pump 600, a photodiode array detector 2996 and an ELSD detector 2420 (Waters SAS, Saint-Quentin-en-Yvelines, France). XAD resin (AMBERLITE™ *XAD*™*16HP* N) was purchased from DOW (DOW France SAS, Saint-Denis, France).

### 4.2. Invertebrate Collection, Transport and Experimental Design

*Anemonia sulcata* individuals (two years old, adult size of 30 g wet weight per unit) were selected from the cultivation facilities of iMare Natural (www.imarenatural.com). *Crambe crambe* individuals (10 to 15 cm length) were carefully collected with their supporting stones from Salobreña, Granada, Spain, at a depth of 8 m, in June 2017 (diving site La Caleta de Salobreña, N 36. 74419, W −3.602065). Sponges were harvested just before starting the experiments. *Crambe crambe* individuals were introduced into plastic bags and kept immersed in seawater. Once on board, plastic bags were introduced in isothermal box, with no aeration and no sunlight exposure.

Transportation from the collection site to iMare facilities took about 2 h. The temperature of the seawater was 24 °C and the salinity was 37‰.

The aquarium experiments were implemented in 35 L aquariums equipped with an 800 L/h submersible pump and a 3 W led light lamp. The pump ensured the continuous circulation of the aquarium water into the trapping filters during 48 h. Each aquarium was equipped with two filters, the first one containing 50 g of silica sand and the second 50 g of Amberlite XAD-16. During the experiment, the invertebrates were not fed and there were no other additives. The experimental system was designed to simulate the environmental conditions of the Summer season, including a high temperature between 25–28 °C and a Summer photoperiod of 12L:12D.

Four aquariums were set up:

- Aquarium 1: was a control without invertebrates,
- Aquarium 2: contained 20 *Anemonia sulcata*
- Aquarium 3: contained *Crambe crambe* individuals covering in total an average surface of 250 to 300 cm^2^.
- Aquarium 4: contained 20 *Anemonia sulcata* and *Crambe crambe* individuals covering in total 250 to 300 cm^2^.

After 48 h, the filters containing the Amberlite XAD-16 resin were recovered and frozen without any treatment until extraction and chemical analysis.

### 4.3. Isolation of Compounds and Struture Elucidation

The Amberlite XAD-16 resin (≈50 g) was poured in a Buchner funnel equipped with a paper filter and washed with 3 × 100 mL of distilled water. The resins were dried under vacuum and transferred to an Erlenmeyer. The compounds trapped by the resin were eluted by a gentle shaking with 3 × 100 mL of ethyl acetate (EtOAc) followed by 3 × 100 mL of methanol (MeOH). The EtOAc and the MeOH fractions were evaporated in vacuo weighed and analyzed by TLC and HPLC. Aquariums containing a single invertebrate species as well as the control aquarium without invertebrates gave extracts weights ranging from 15 to 20 mg. TLC and HPLC analysis gave flat chromatograms without any exploitable signals.

The XAD-16 resin recovered from the aquarium in which *Crambe crambe* individuals were added to *Anemonia sulcata* (Aq 4) yielded 22 mg of crude EtOAc extract and 160 mg of MeOH extract. The EtOAc extract did not show any exploitable signals after TLC and HPLC analysis. The MeOH extract (160 mg) was submitted to flash chromatography on a Combiflash Companion using a Redisep 12 g silica column eluted with a gradient of methylene dichloromethane (DCM) and MeOH. Based on the TLC and HPLC profiles, 17 fractions were pooled and submitted to single peak preparative HPLC purification. The pure compounds were further analyzed by LC-MS and the peaks with similar mass and chromatographic data were pooled to obtain the maximum quantities for structural elucidation. Thereby we isolated 1.5 mg of crambescidin 359 (**1**), 1.1 mg of crambescidin acid (**2**), 1.1 mg of crambescidin 401 (**3**), 1.6 mg of crambescin B 281 (**4**) and 1.8 mg of crambescin B 253 (**5**).

*Crambescidin 359* (**1**), *α*^25^_D_ − 135.0 (*c* 0.2, MeOH). IR (neat) ν_max_ 2967, 2933, 1620, 1449, 1344, 1010, 847, 755. NMR data Table S30 (supporting information); HR-ESI-MS for C_21_H_33_N_3_O_2_ (*m/z* 360.2651 [M+H]^+^) (calcd for C_21_H_34_N_3_O_2_, 360.2573).

*Crambescidin acid* (**2**), *α*^25^_D_ − 45.0 (*c* 0.06, CHCl_3_). IR (neat) ν_max_ 3230, 2961, 2931, 1622, 1446, 1398, 1342, 1047, 1011, 755, 716. NMR data, Spectra S6 and S7 and Table S30 (supporting information); HR-ESI-MS for C_22_H_33_N_3_O_4_ (*m/z* 404.2549 [M+H]^+^) (calcd for C_22_H_34_N_3_O_4_, 404.2471).

*Crambescidin 401* (**3**), *α*^25^_D_ − 48.6 (*c* 0.07, CHCl_3_). IR (neat) ν_max_ 3358, 2963, 2929, 1620, 1580, 1360, 1053, 1026, 754 NMR data, Spectra S10 to S14 and Table S30 (supporting information); HR-ESI-MS for C_22_H_31_N_3_O_4_ (*m/z* 402.2393 [M+H]^+^) (calcd for C_22_H_32_N_3_O_4_, 402.2315).

*Crambescin B 281* (**4**), *α*^25^_D_ + 103.8 (*c* 0.1, CHCl_3_). IR (neat) ν_max_ 3236, 3067, 2924, 2855, 2776, 1650, 1610, 1582, 1464, 1346, 1027, 759.NMR data, Spectra S17 to S21 and Table S31 (supporting information); HR-ESI-MS for C_16_H_32_N_3_O (*m/z* 282.2544 [M+H]^+^) (calcd for C_16_H_32_N_3_O, 282.2545).

*Crambescin B 253* (**5**), *α*^25^_D_ + 124.2 (*c* 0.05, CHCl_3_). IR (neat) ν_max_ 3224, 3062, 2956, 2856, 1650, 1609, 1581, 1462, 1346, 1028, 761. NMR data, Spectra S24 to S28 and Table S31 (supporting information); HR-ESI-MS for C_14_H_28_N_3_O (*m/z* 254.2227 [M+H]^+^) (calcd for C_14_H_28_N_3_O_1_, 254.2232).

## 5. Patents

Somartex^®^ (Self Operating MARine Trapping Extractor) is a unique technology inspired by the results of this paper. The technology was built to allow the trapping of the compounds released by an invertebrate or a community of invertebrates directly into their marine natural habitat. It is formed by (1) a cover cone with various diameters and heights which delimits the area of interest, (2) a submerged pump that drives the water around the invertebrate into the cone. The water is expected to contain valuable compounds secreted by the invertebrate (3) a trapping module in which the water is brought into contact with one or more trapping matrix which also increases the concentration of the compounds. The trapping material is then recovered after a defined period of contact and the target compounds are desorbed. The power for the pump can be supplied by renewable solar energy, associated with an accumulator to allow continuous and autonomous operations. Solar panels will be installed on a 2.4 m diameter floating solid platform. The operator can reach the platform (using a dinghy) to carry out any maintenance, recover/replace the trapping material or move the equipment to another area.

## Figures and Tables

**Table 1:** ^1^H NMR (600 MHz, cryoprobe) and ^13^C NMR (150 MHz) data for crambescidin acid (**2**) and crambescidin 401 (**3**) in CD_3_OD.

	Crambescidin Acid (2)			Crambescidin 401 (3)		
position	δ_C_, type	δ_H_, mult, *J* in Hz	HMBC	δ_C_, type	δ_H_, mult, *J* in Hz	HMBC
1	10.7, CH_3_	0.80; t (7.3)	2, 3	10.9, CH_3_	0.88; t (7.4)	2, 3
2	30.8, CH_2_	1.54, m; 1.47, m	-	30.2, CH_2_	1.54, m; 1.46, m	-
3	72.2, CH	4.34, br m	-	72.2, CH	4.56, br m	-
4	134.5, CH	5.51, br d (11.2)	2, 3, 6	134.3, CH	5.54, br d (11.1)	3, 6
5	131.7, CH	5.72, br m	3, 6, 7	131.4, CH	5.76, br m	3, 6, 7
6	24.6, CH_2_	2.15, m; 2.43, m	4, 5, 7, 8	24.5, CH_2_	2.15, m; 2.43, m	4, 5, 7, 8
7	37.4, CH_2_	2.02, m; 2.24 m	5, 6, 8, 9	37.8, CH_2_	2.02, m; 2.61 br t (13.4)	5, 6, 8, 9
8	85.2, C	-	-	86.6, C	-	-
9	37.8, CH_2_	1.50 m; 2.53 dd (12.9, 3.3)	8, 10, 11	35.4, CH_2_	1.6 t (12.9); 2.81 dd (12.9, 3.3)	8, 10, 11
10	55.8, CH	4.00, m	9	60.1, CH	4.82, m	-
11	30.9, CH_2_	1.74, m; 2.32, m	-	30.5, CH_2_	2.06, m; 2.68, m	12, 13
12	28.5, CH_2_	2.06, m; 2.25, m	10, 11,13	31.4, CH_2_	3.38, m	10, 11,13
13	54.9, CH	4.21, m	12, 14, 22	160.7, C	-	-
14	54.1, CH	2.68, d (4.7)	13, 15, 16, 22	123.6, C	-	-
15	82.8, C	-	-	86.6, C	-	-
16	32.8, CH_2_	1.27, m; 1.68, m	-	37.4, CH_2_	3.01–3.08, br m	14, 17, 18, 22
17	19.8, CH_2_	1.80, m; 1.87, m	15, 16	25.1, CH_2_	1.80, m; 1.87, m	16, 22
18	34.5, CH_2_	1.48, br m	-	39.5, CH_2_	1.48, br m	-
19	67.8, CH	3.81, m	17, 18	68.0, CH	3.74, m	17
20	22.1, CH_3_	1.08, d (6.2)	18, 19	21.9, CH_3_	1.14, d (6.2)	18, 19
21	150.8, C	-	-	151.3, C	-	-
22	175.1, C	-	-	179.5, C	-	-

**Table 2:** ^1^H NMR (500 MHz) and ^13^C NMR (125 MHz) Data for crambescin B 281 (**4**) and crambescin B 253 (**5**) in CD_3_OD.

	4			5		
position	δ_C_, type	δ_H_, mult, *J* in Hz	HMBC	δ_C_, type	δ_H_, mult, *J* in Hz	HMBC
1	154.4, C	-	-	154.4, C	-	-
2	88.0, C	-	-	88.0, C	-	-
3	67.3, CH_2_	3.86, 3.98; dd (12.8, 6.5)	2, 4	67.3, CH_2_	3.86, 3.98; dd (12.8, 6.5)	2, 4
4	54.7, CH_2_	1.97, 2.22; m	-	54.7, CH_2_	1.97, 2.22; m	-
5	36.8, CH_2_	1.91, 2.14; m	2, 4	36.8, CH_2_	1.91, 2.14; m	2, 4
6	37.7, CH_2_	1.62, 1.93; m	2, 7, 8, 9	37.7, CH_2_	1.62, 1.93; m	2, 7, 8, 9
7	47.1, CH	3.59; m	-	47.1, CH	3.59; m	-
8	34.9, CH_2_	1.49, 1.63; m	7	34.9, CH_2_	1.49, 1.63; m	7
9	25.1, CH_2_	1.22–1.14; m	-	25.1, CH_2_	1.22–1.13; m	-
10	29.5, CH_2_	1.22–1.14; m		29.4, CH_2_	1.22–1.13; m	-
11	29.5, CH_2_	1.22–1.14; m		29.2, CH_2_	1.22–1.13; m	-
12	29.4, CH_2_	1.22–1.14; m	-	38.1, CH_2_	1.22–1.13; m	-
13	29.3, CH_2_	1.22–1.14; m	-	22.9, CH_2_	1.22–1.13; m	-
14	38.1, CH_2_	1.22–1.14; m	-	14.1, CH_3_	0.87; t (7.1)	12, 13
15	22.9, CH_2_	1.22–1.14; m	-			
16	14.1, CH_3_	0.87; t (7.1)	14, 15			

## Supplementary Materials

The following are available online at http://www.mdpi.com/1660-3397/16/5/152/s1.
