# Supplementary material for: Innovative Approach to Sustainable Marine Invertebrate Chemistry and a Scale-Up Technology for Open Marine Ecosystems
Source: Mar Drugs. 2018 May 6;16(5):152. doi: 10.3390/md16050152 (PMC5983283; doi:10.3390/md16050152)
Supplement: Supplementary file 1 [file marinedrugs-16-00152-s001.pdf]

Innovative Approach to Sustainable Marine Invertebrate Chemistry and a Scale-Up Technology for Open Marine Ecosystems.

Pinelopi Vlachou [2], Géraldine Le Goff [1], Carolina Alonso [3], Pedro A. Álvarez [3], Jean-François Gallard [1], Nikolas Fokialakis [2] and Jamal Ouazzani [1,*]

1 Institut de Chimie des Substances Naturelles ICSN, Centre National de la Recherche Scientifique, Avenue de la Terrasse 91198, Gif-sur-Yvette, France; geraldine.legoff@cnrs.fr (G.L.G); Jean-francois.Gallard@cnrs.fr (J.F.G), jamal.ouazzani@cnrs.fr (J.O)

2 Department of Pharmacognosy & Natural Products Chemistry, Faculty of Pharmacy, National and Kapodistrian University of Athens, Athens 15771 Greece; pvlachou@pharm.uoa.gr (P.V); fokialakis@pharm.uoa.gr (N.F)

3 iMARE natural S.L. Avda. Habana, 10, 18600, Motril, Spain ; carolina.imare@gmail.com (C.A); pedro.imare@gmail.com (P.A.A)

Elemental Composition Report

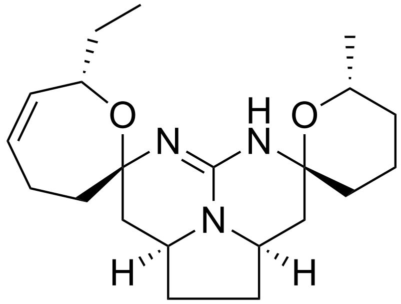

crambescidin 359 (**1**)

Single Mass Analysis

Tolerance = 20.0 PPM / DBE: min = -1.5, max = 100.0
Element prediction: Off
Number of isotope peaks used for i-FIT = 9

Monoisotopic Mass, Even Electron Ions
460 formula(e) evaluated with 6 results within limits (all results (up to 1000) for each mass)
Elements Used:
C: 0-50 H: 0-100 N: 0-10 O: 0-10

OUAZZANI_glegoff56-1 23 (0.608) Cm (21:26)

1: TOF MS ES+
8.30e+004

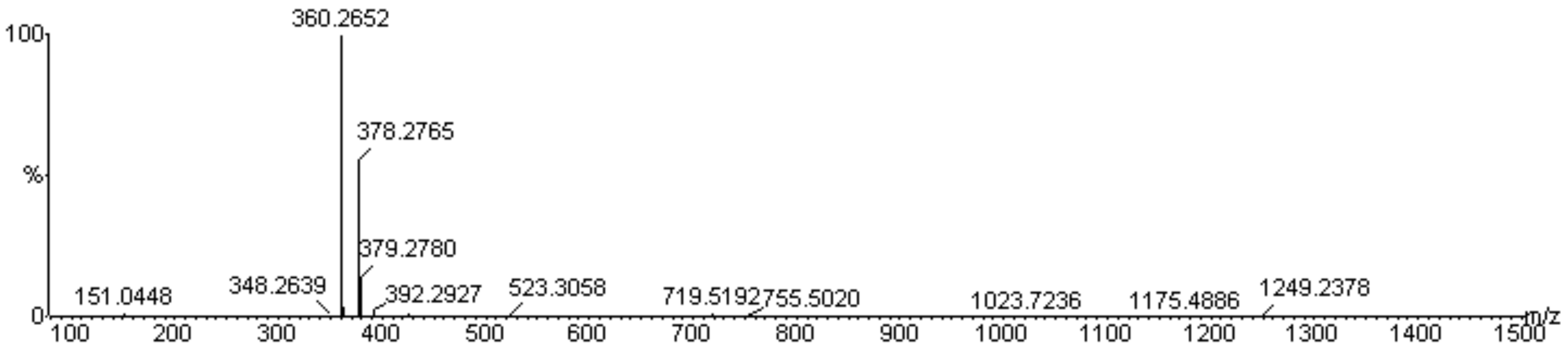

Minimum:				-1.5			
Maximum:		5.0	20.0	100.0			
Mass	Calc. Mass	mDa	PPM	DBE	i-FIT	i-FIT (Norm)	Formula
360.2652	360.2651	0.1	0.3	6.5	999.5	0.0	C21 H34 N3 O2
	360.2624	2.8	7.8	7.5	1006.0	6.4	C17 H30 N9
	360.2683	-3.1	-8.6	-1.5	1014.2	14.7	C10 H34 N9 O5
	360.2691	-3.9	-10.8	10.5	1007.1	7.5	C26 H34 N
	360.2611	4.1	11.4	2.5	1007.5	8.0	C16 H34 N5 O4
	360.2723	-7.1	-19.7	2.5	1008.8	9.3	C15 H34 N7 O3

S1- crambescidin 359 (**1**) HRESIMS

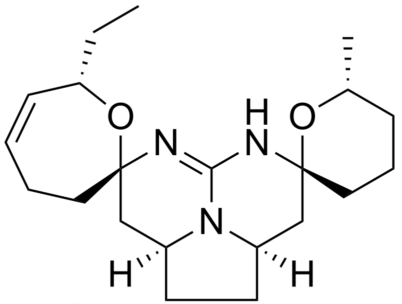

crambescidin 359 (**1**)

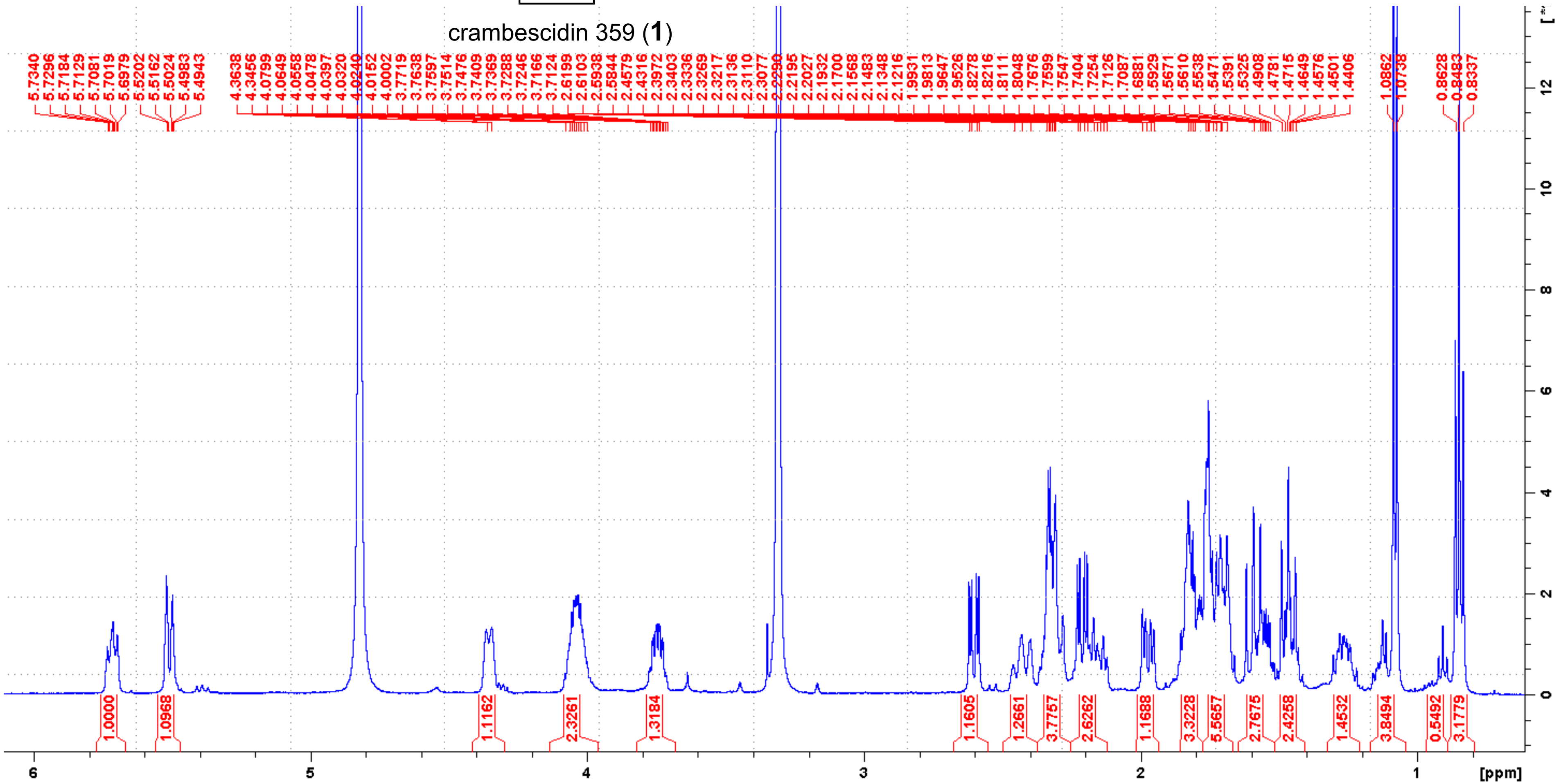

S2- crambescidin 359 (**1**) ^{1}H-NMR spectrum (500 MHz, MeOD)

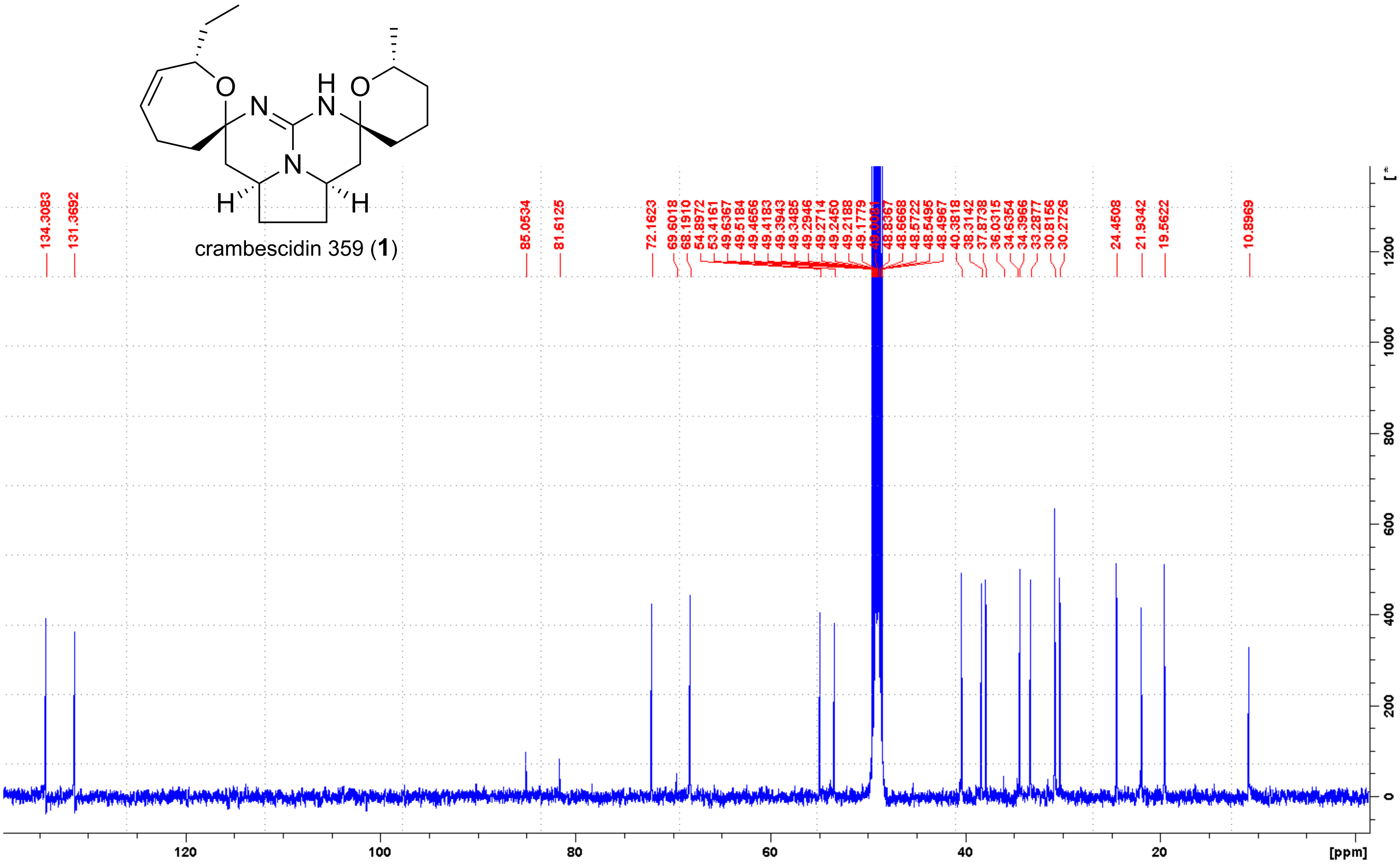

S3- crambescidin 359 (**1**) ^{13}C-NMR spectrum (125 MHz, MeOD)

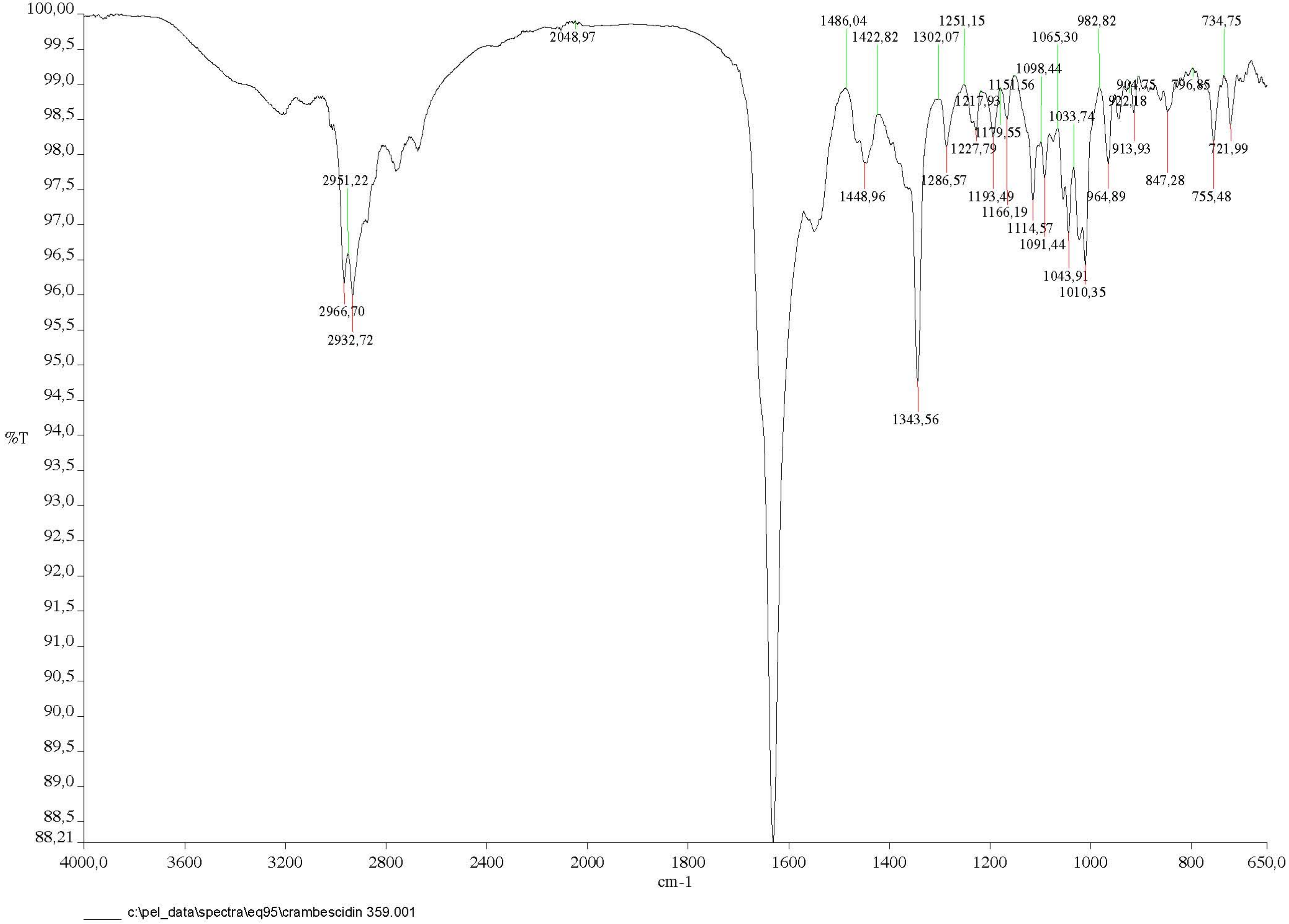

S4- crambescidin 359 (**1**) IR spectrum

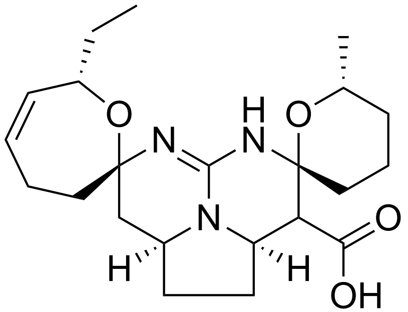

crambescidin acid (**2**)

Elemental Composition Report

Single Mass Analysis

Tolerance = 10.0 PPM / DBE: min = -1.5, max = 100.0
Element prediction: Off
Number of isotope peaks used for i-FIT = 9

Monoisotopic Mass, Even Electron Ions
526 formula(e) evaluated with 5 results within limits (all results (up to 1000) for each mass)
Elements Used:
C: 0-50 H: 0-100 N: 0-10 O: 0-10

OUAZZANI_glegoff58-2 24 (0.625) Cm (20:28-39:65x2.000)

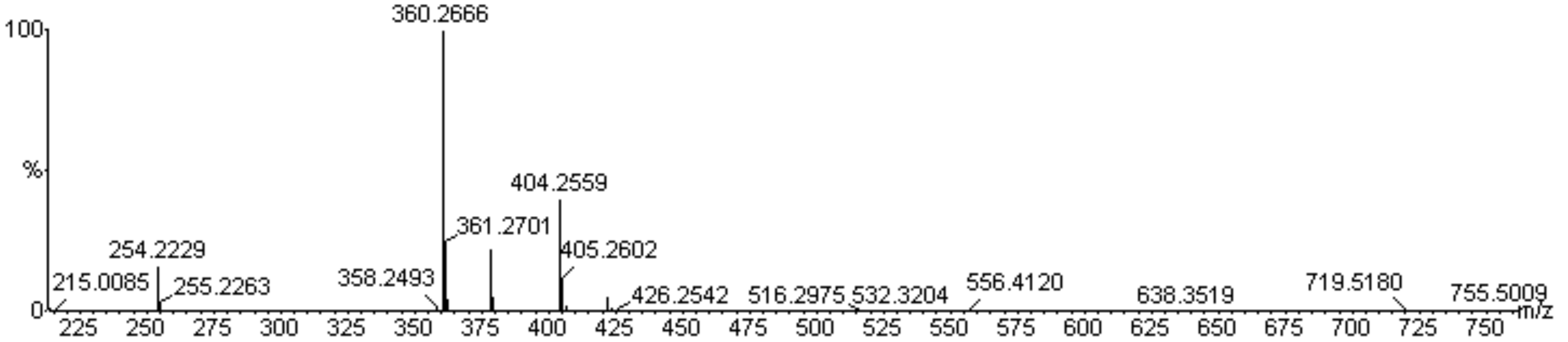

Minimum:				-1.5			
Maximum:		5.0	10.0	100.0			

Mass	Calc. Mass	mDa	PPM	DBE	i-FIT	i-FIT (Norm)	Formula
404.2559	404.2563	-0.4	-1.0	12.5	292.4	3.0	C23 H30 N7
	404.2549	1.0	2.5	7.5	289.5	0.1	C22 H34 N3 O4
	404.2581	-2.2	-5.4	-0.5	306.9	17.5	C11 H34 N9 O7
	404.2590	-3.1	-7.7	11.5	293.7	4.3	C27 H34 N O2
	404.2522	3.7	9.2	8.5	298.9	9.5	C18 H30 N9 O2

S5- crambescidin acid (**2**) HRESIMS

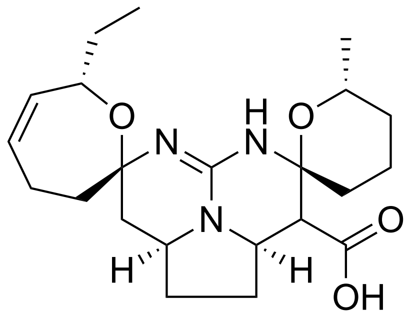

crambescidin acid (**2**)

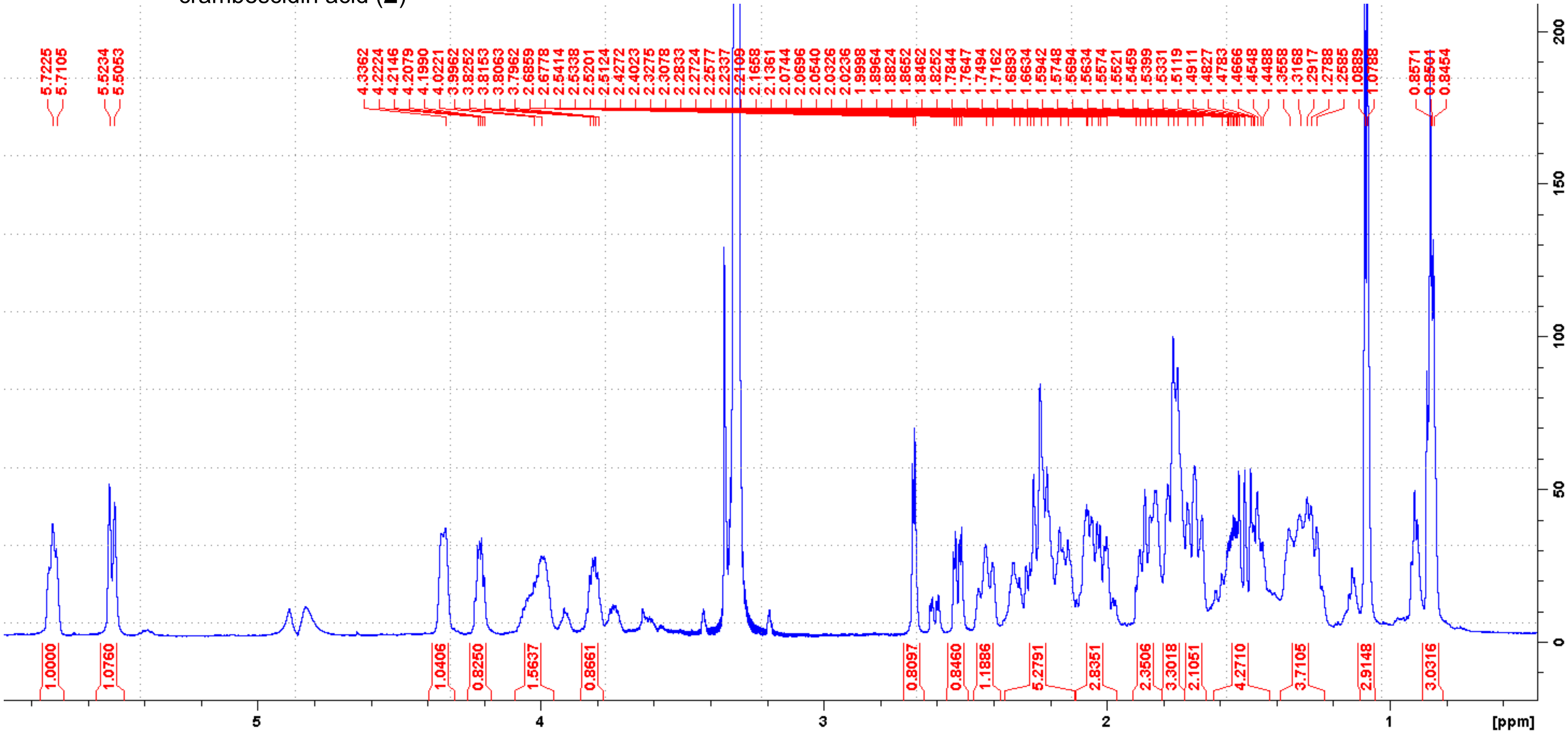

S6- crambescidin acid (**2**) ^{1}H-NMR spectrum (600 MHz, MeOD)

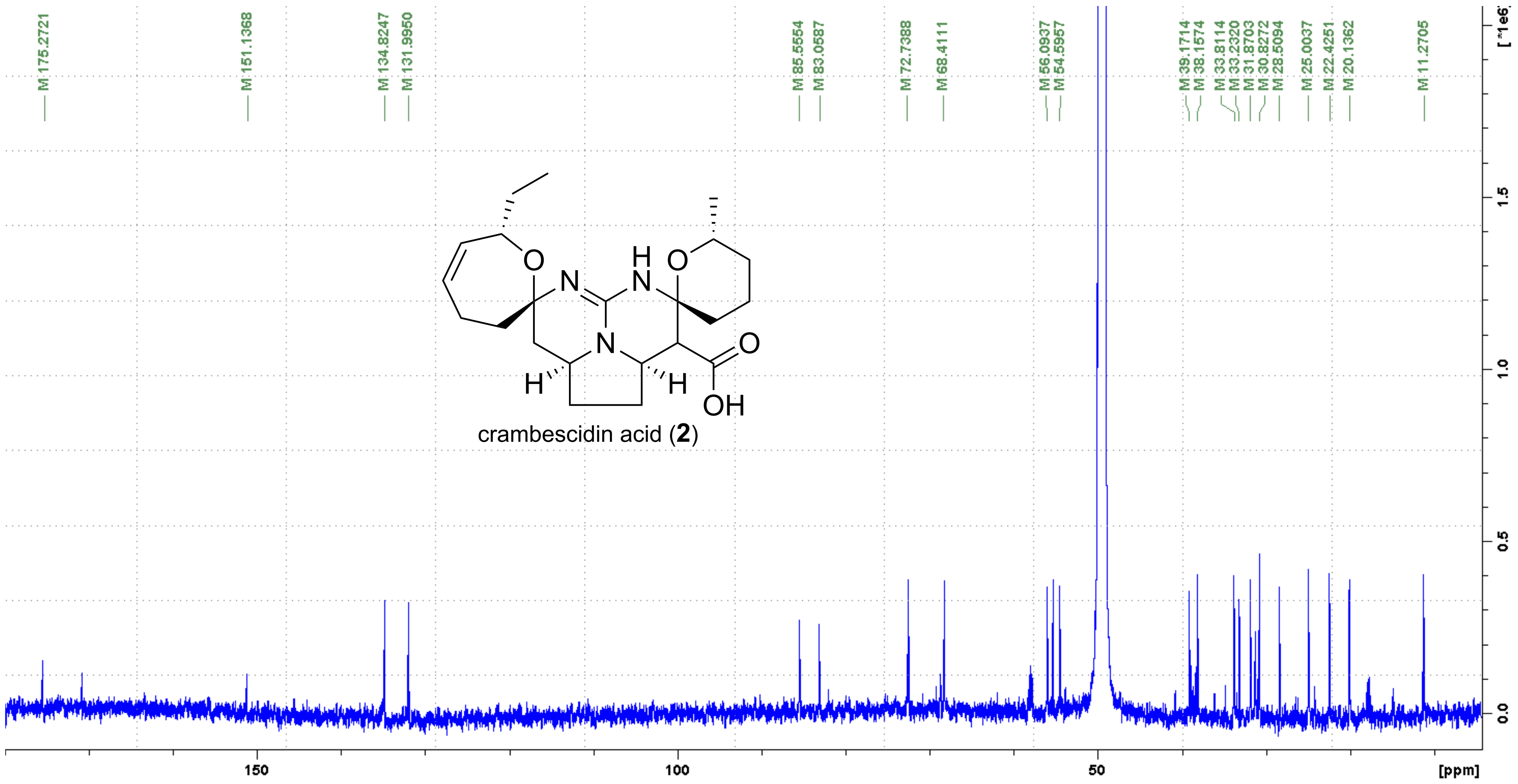

S7- crambescidin acid (**2**) ^{13}C-NMR spectrum (150 MHz, MeOD)

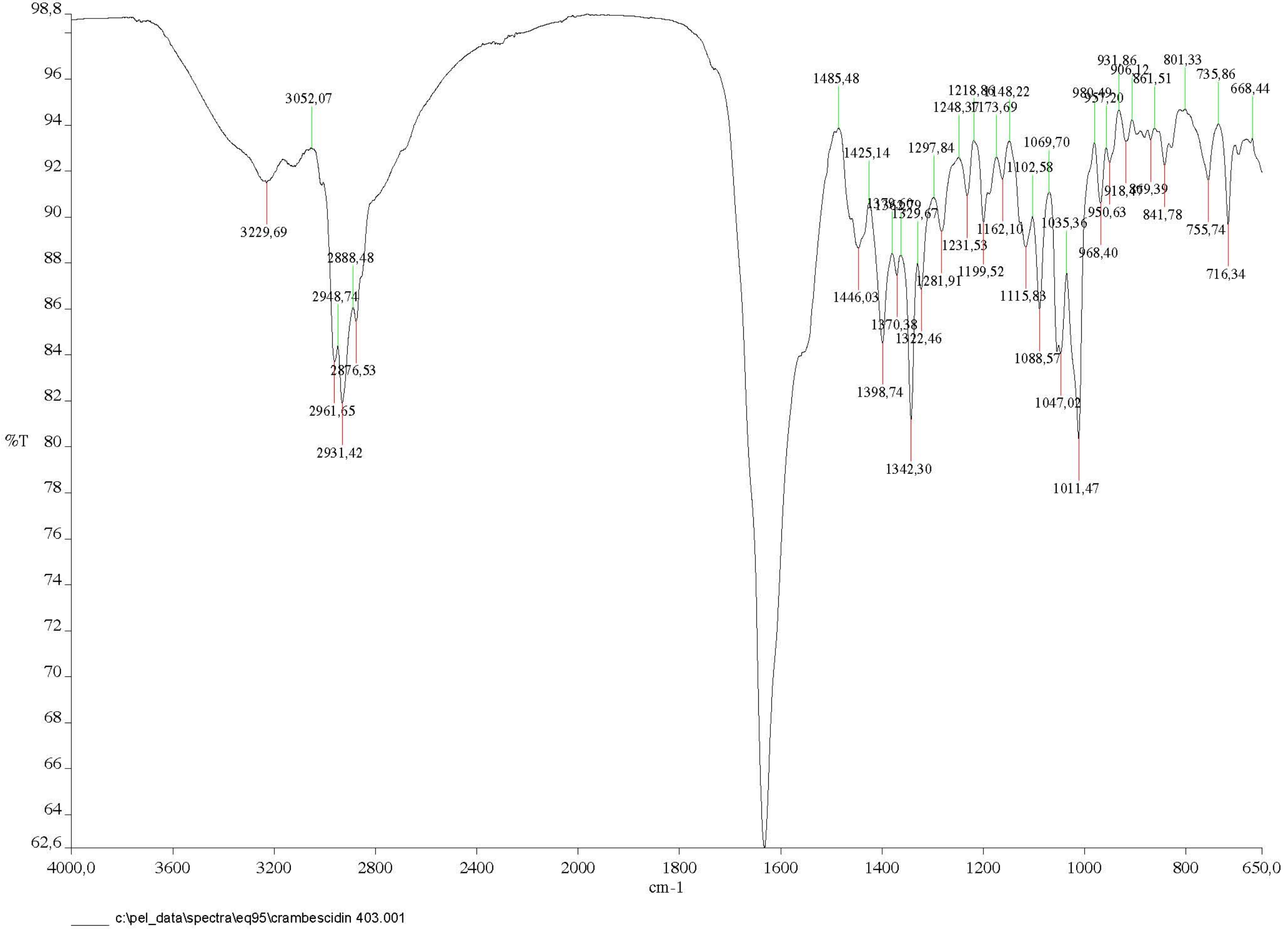

S8- crambescidin acid (**2**) IR spectrum

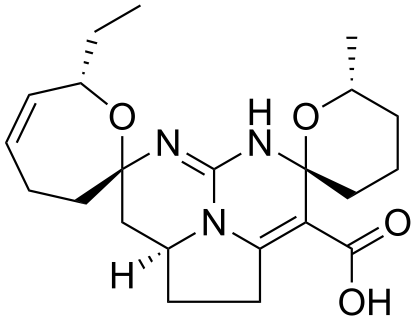

crambescidin 401 (**3**)

Elemental Composition Report

Single Mass Analysis

Tolerance = 10.0 PPM / DBE: min = -1.5, max = 100.0
Element prediction: Off
Number of isotope peaks used for i-FIT = 9

Monoisotopic Mass, Even Electron Ions
519 formula(e) evaluated with 4 results within limits (all results (up to 1000) for each mass)
Elements Used:
C: 0-50 H: 0-100 N: 0-10 O: 0-10

OUAZZANI_glegoff58-1 23 (0.607) Cm (18:28-34:67x2.000)

1: TOF MS ES+
4.44e+004

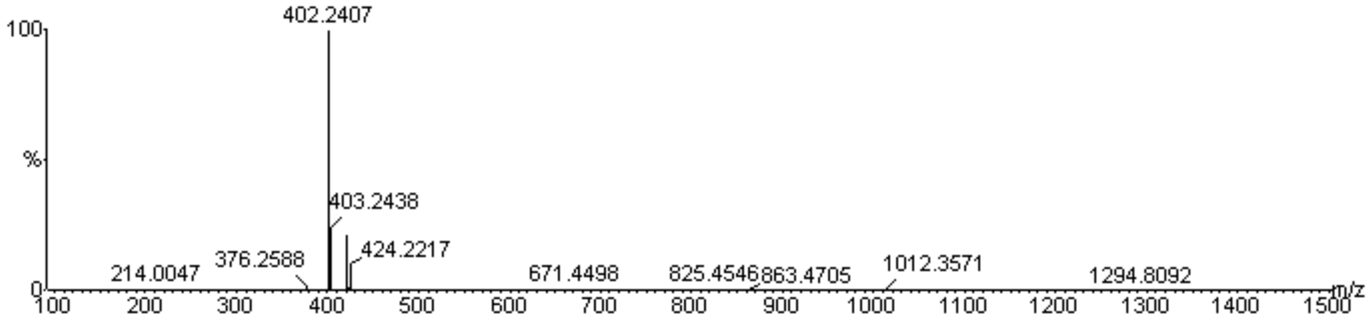

Minimum:				-1.5			
Maximum:		5.0	10.0	100.0			
Mass	Calc. Mass	mDa	PPM	DBE	i-FIT	i-FIT (Norm)	Formula
402.2407	402.2406	0.1	0.2	13.5	520.2	3.4	C23 H28 N7
	402.2393	1.4	3.5	8.5	516.8	0.0	C22 H32 N3 O4
	402.2425	-1.8	-4.5	0.5	521.2	4.4	C11 H32 N9 O7
	402.2433	-2.6	-6.5	12.5	525.0	8.2	C27 H32 N O2

S9- crambescidin 401 (**3**) HRESIMS

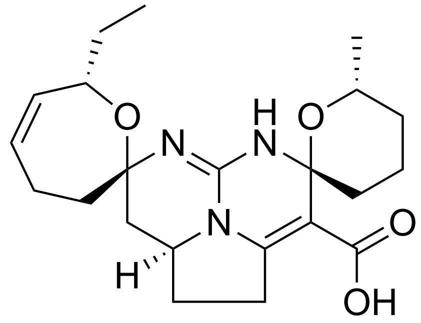

crambescidin 401 (**3**)

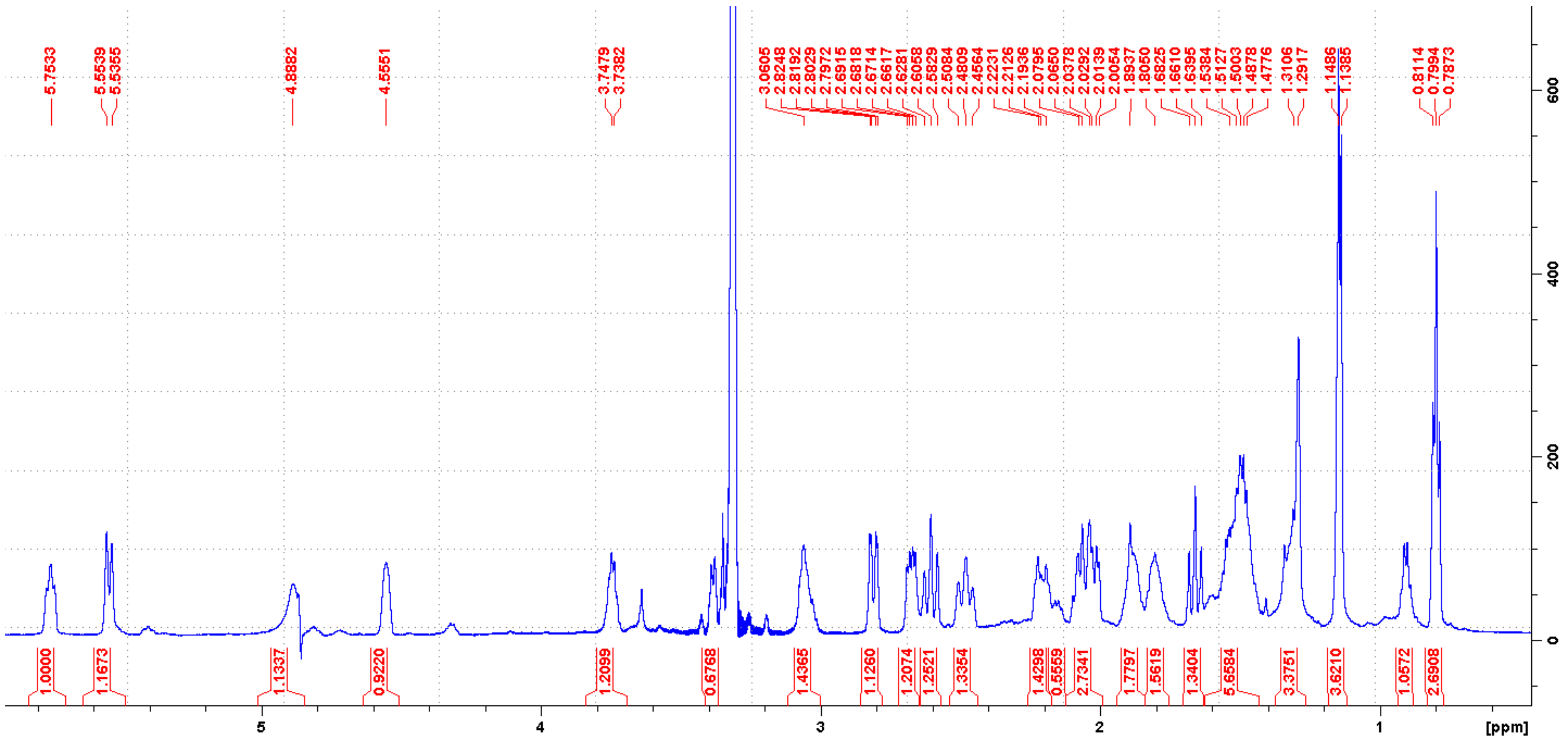

S10- crambescidin 401 (**3**) ^{1}H-NMR spectrum (600 MHz, MeOD)

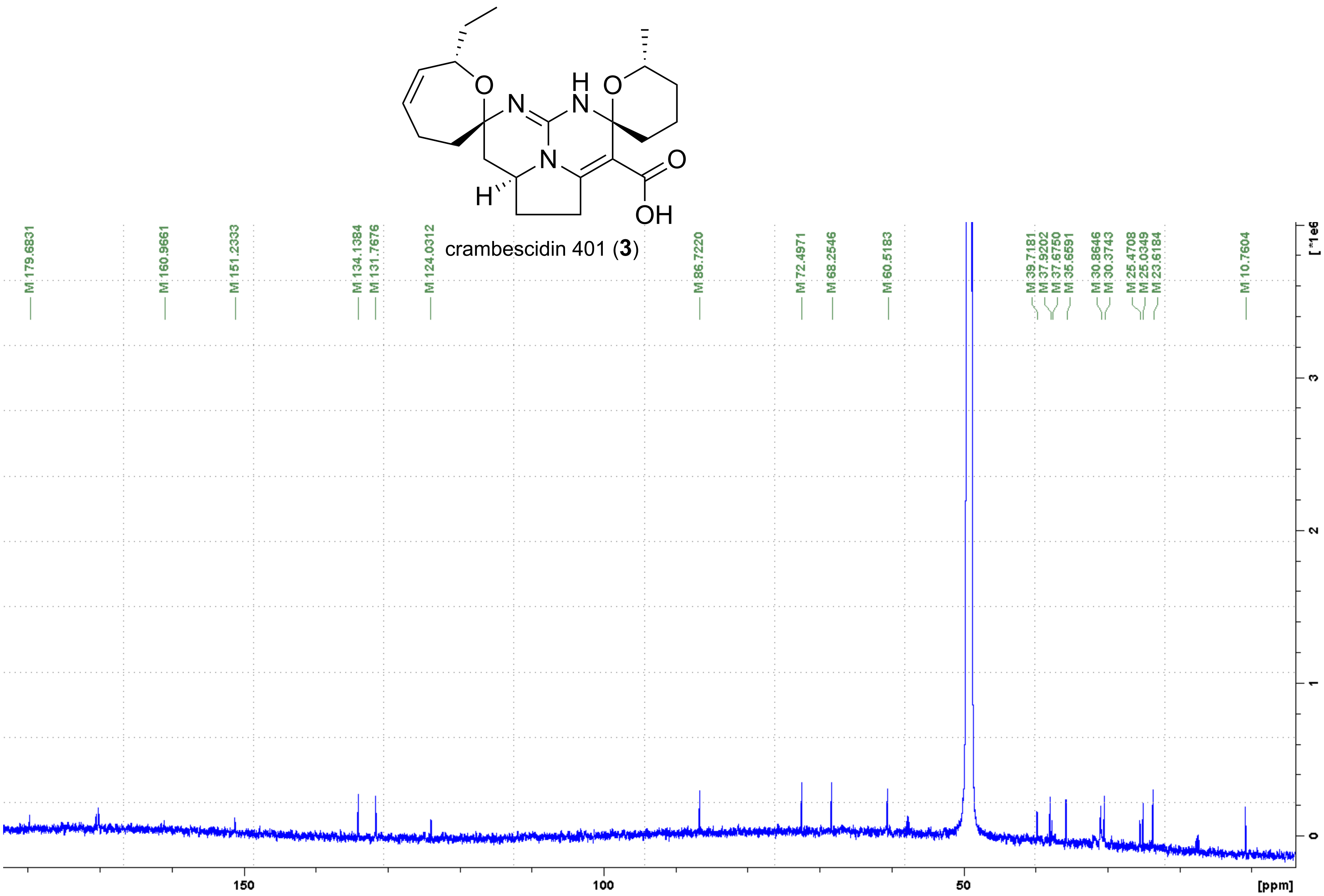

S11- crambescidin 401 (**3**) ^{13}C-NMR spectrum (150 MHz, MeOD)

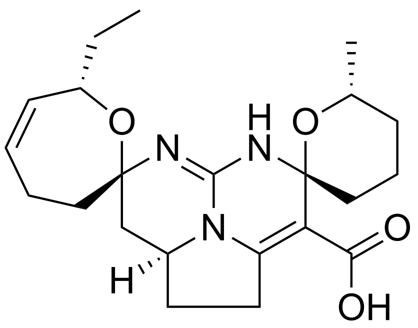

crambescidin 401 (**3**)

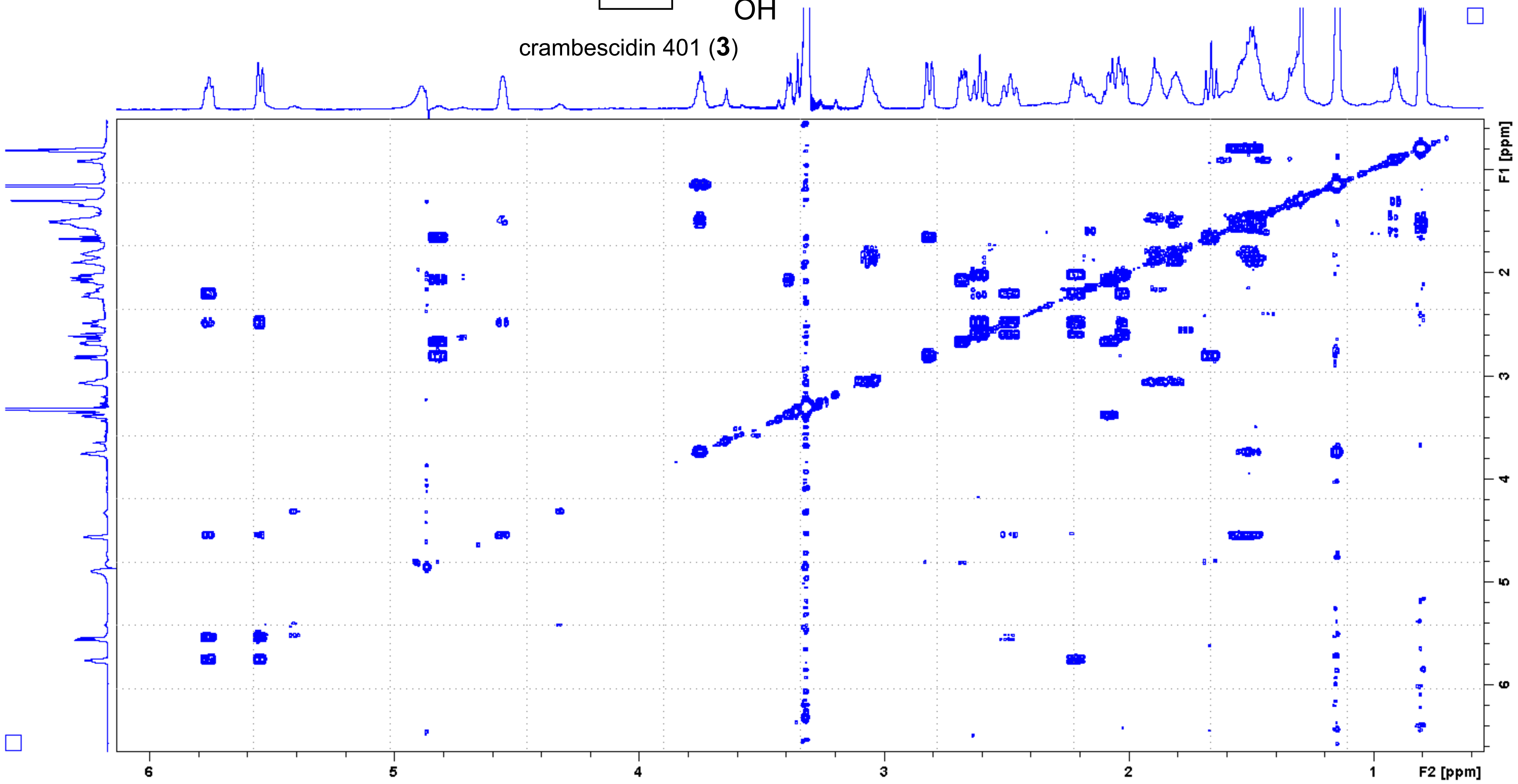

S12- crambescidin 401 (**3**) ^{1}H-^{1}H COSY spectrum (600 MHz, MeOD)

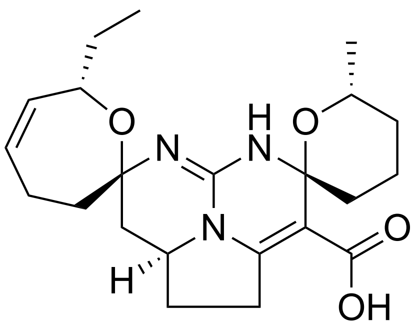

crambescidin 401 (**3**)

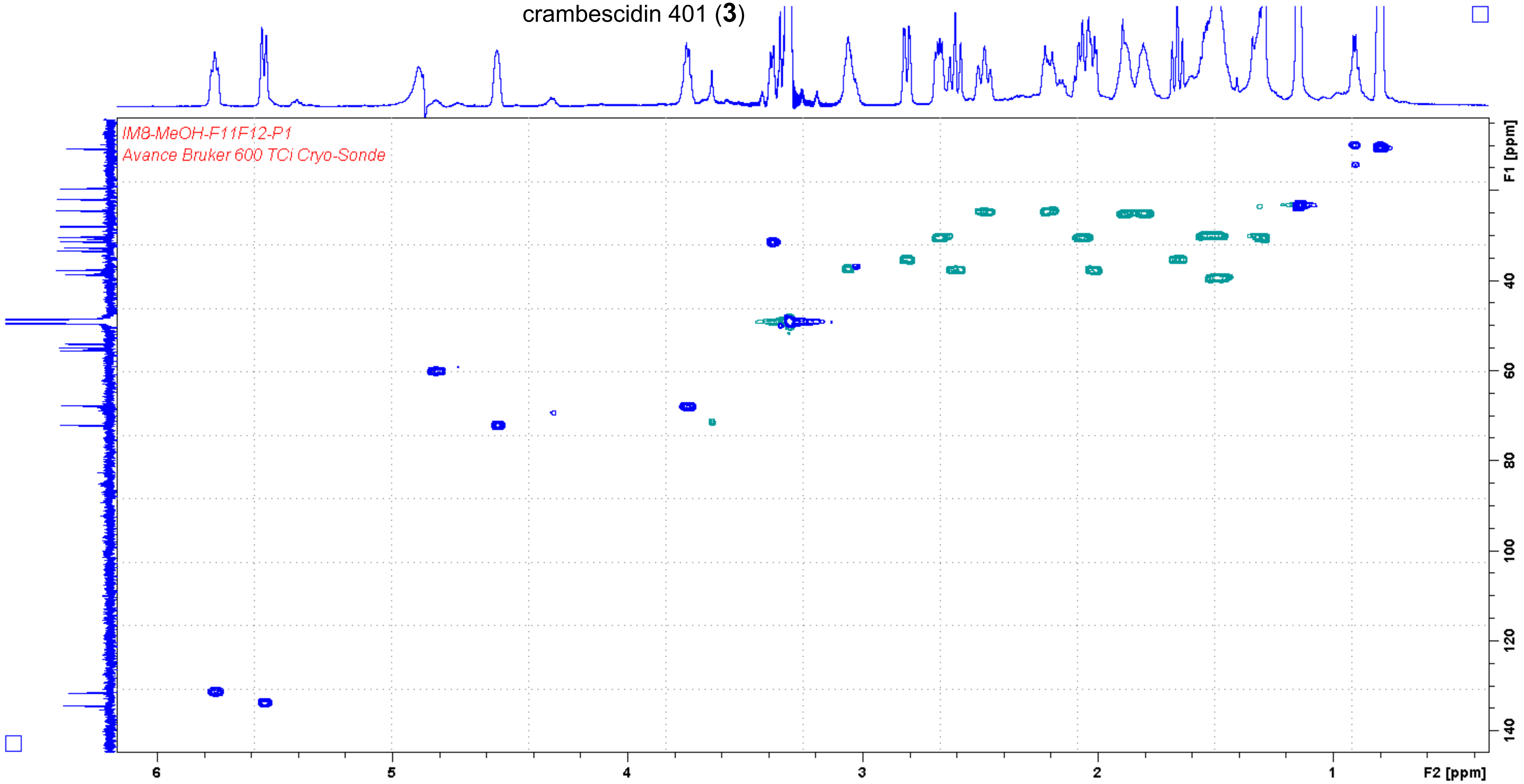

S13- crambescidin 401 (**3**) ^{1}H-^{13}C HSQC spectrum (600 MHz, MeOH)

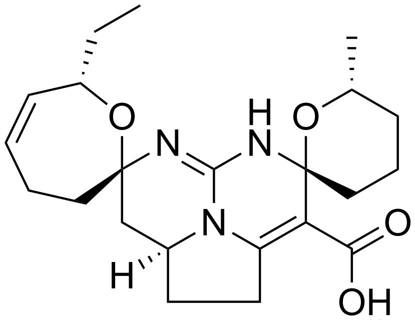

crambescidin 401 (**3**)

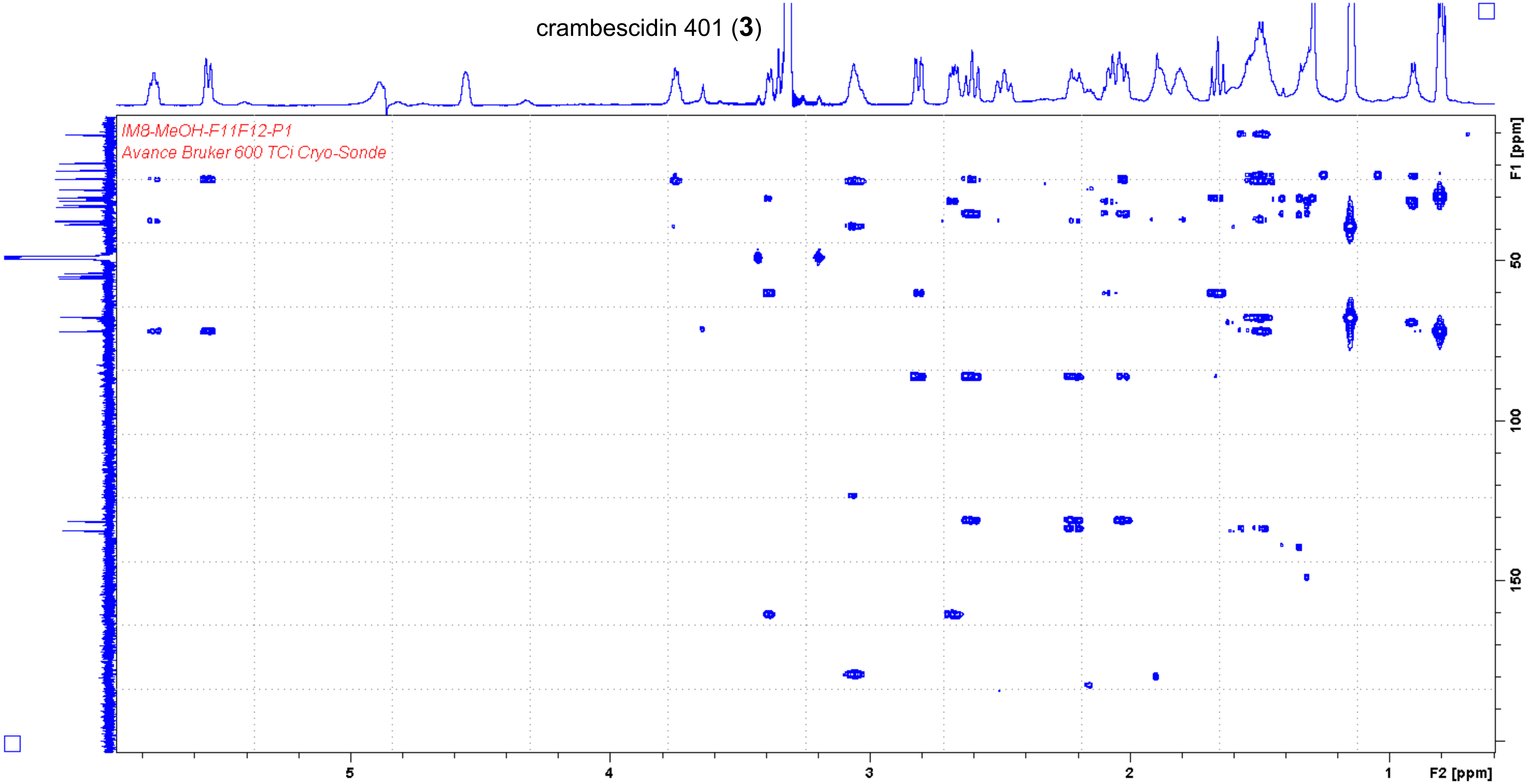

S14- crambescidin 401 (**3**) ^{1}H-^{13}C HMBC spectrum (600 MHz, MeOD)

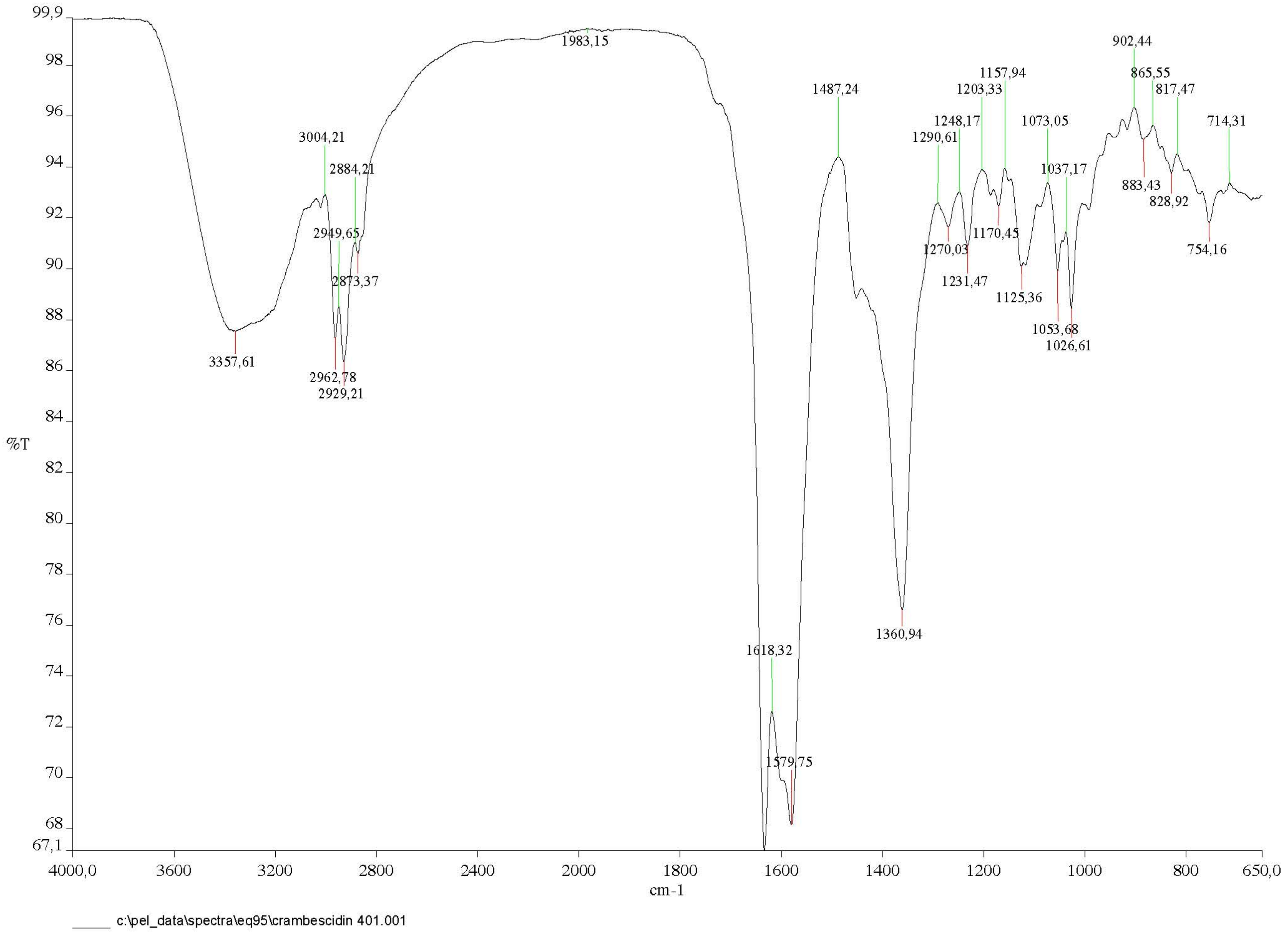

S15- crambescidin 401 (**3**) IR spectrum

Elemental Composition Report

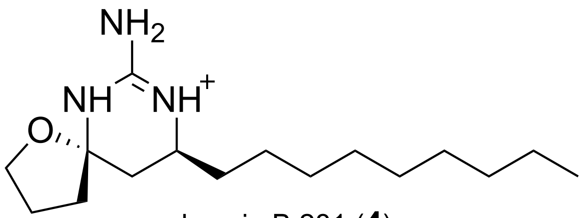

crambescin B 281 (**4**)

Single Mass Analysis

Tolerance = 20.0 PPM / DBE: min = -1.5, max = 100.0
Element prediction: Off
Number of isotope peaks used for i-FIT = 9

Monoisotopic Mass, Even Electron Ions
344 formula(e) evaluated with 2 results within limits (all results (up to 1000) for each mass)
Elements Used:
C: 0-50 H: 0-100 N: 0-10 O: 0-10

OUAZZANI_glegoff57-4 22 (0.590) Cm (19:28-35:70x2.000) 1: TOF MS ES+ 2.26e+005

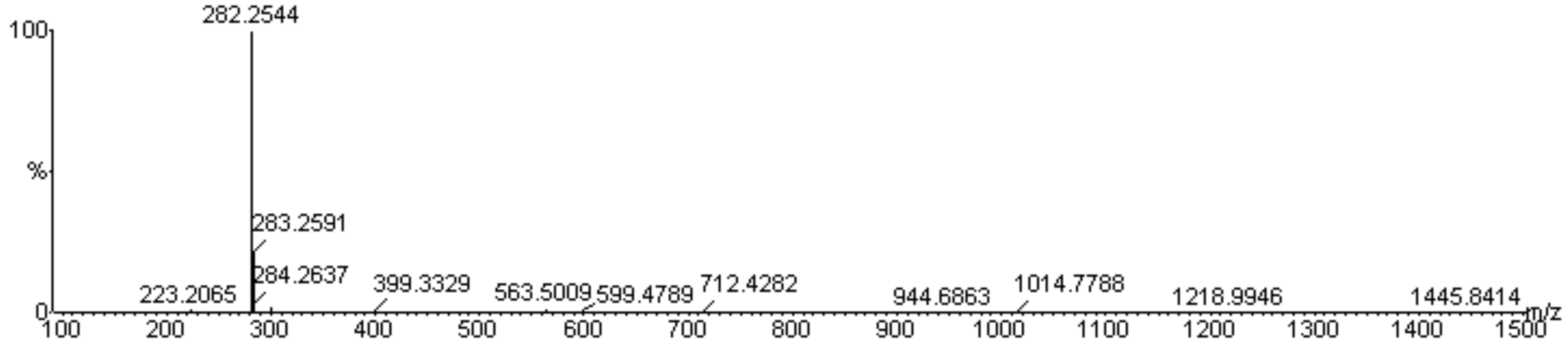

Mass	Calc. Mass	mDa	PPM	DBE	i-FIT	i-FIT (Norm)	Formula
Minimum:				-1.5			
Maximum:		5.0	20.0	100.0			
282.2544	282.2545	-0.1	-0.4	2.5	950.5	0.0	C16 H32 N3 O
	282.2505	3.9	13.8	-1.5	958.8	8.3	C11 H32 N5 O3

S16- crambescin B 281 (**4**) HRESIMS

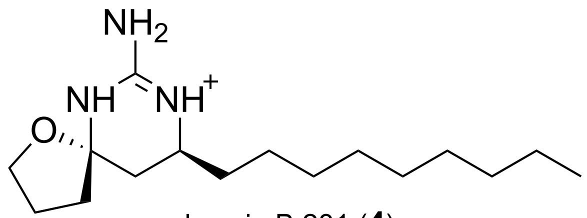

crambescin B 281 (**4**)

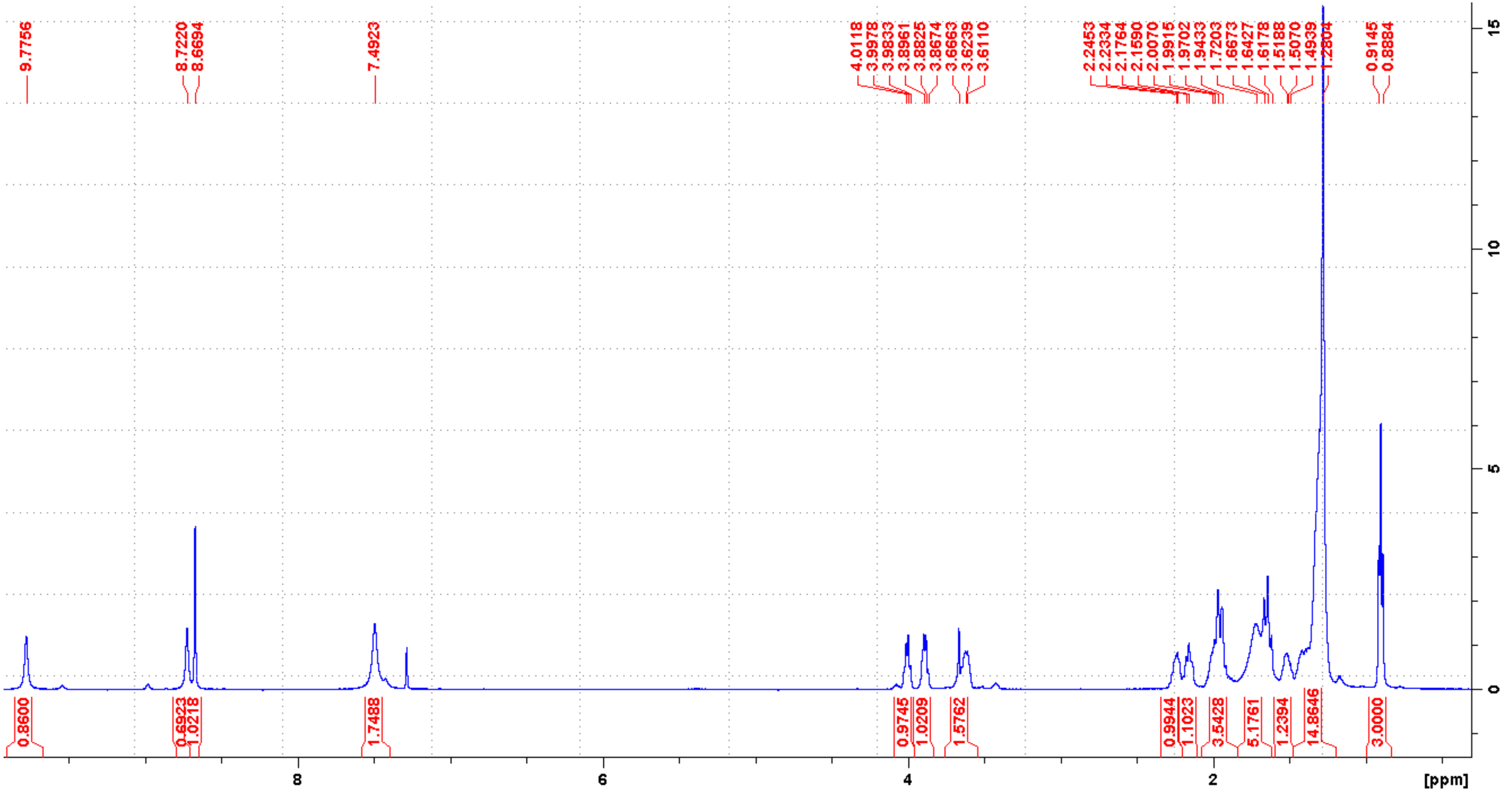

S17- crambescin B 281 (**4**) ^{1}H-NMR spectrum (500 MHz, $CDCl_3$)

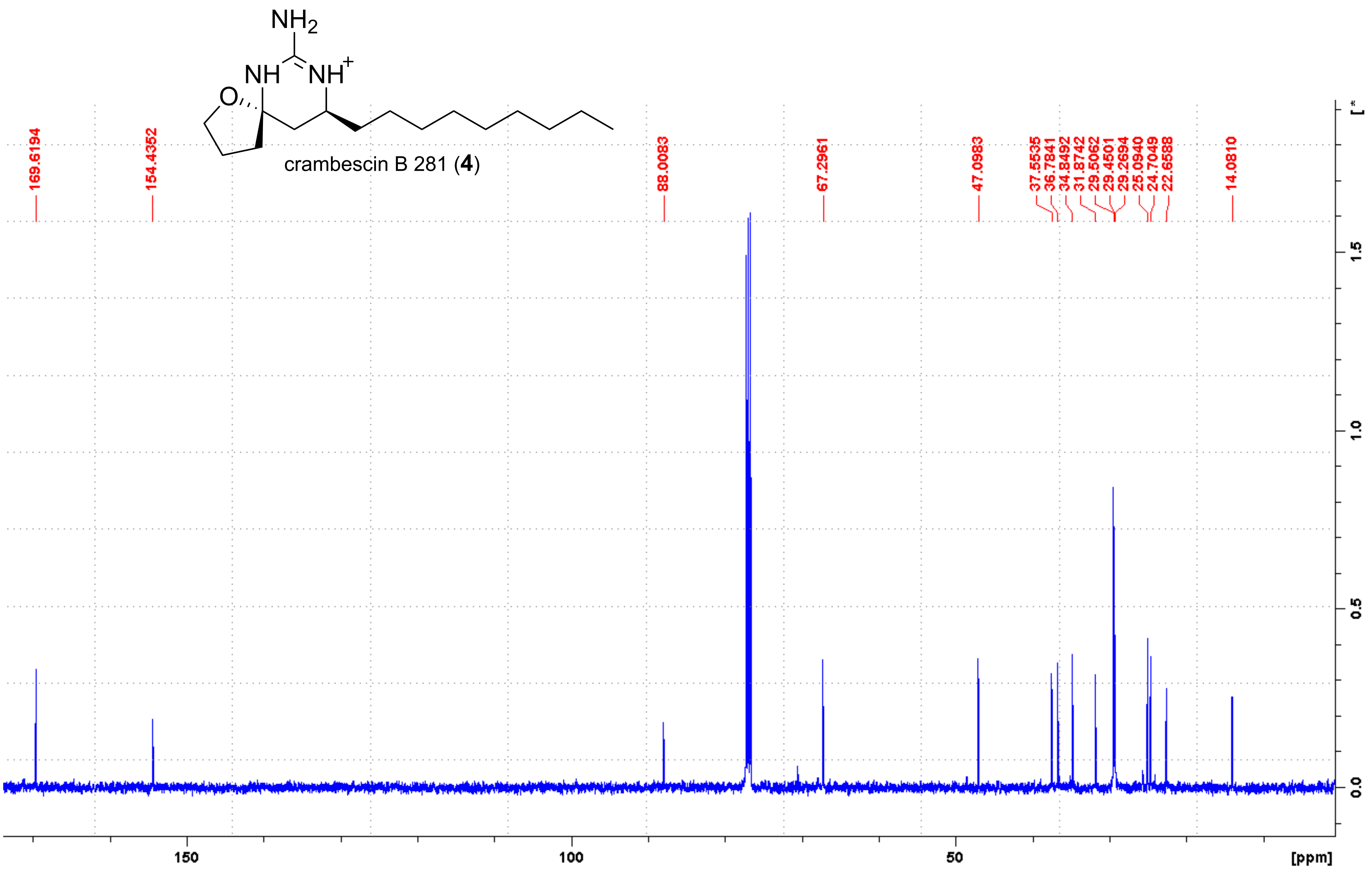

S18- crambescin B 281 (**4**) ^{13}C-NMR spectrum (125 MHz, $CDCl_3$)

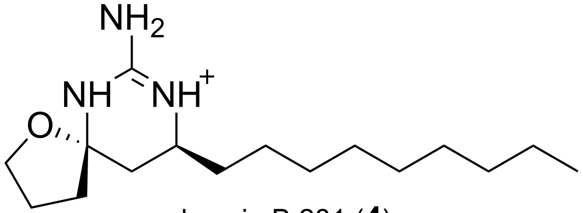

crambescin B 281 (**4**)

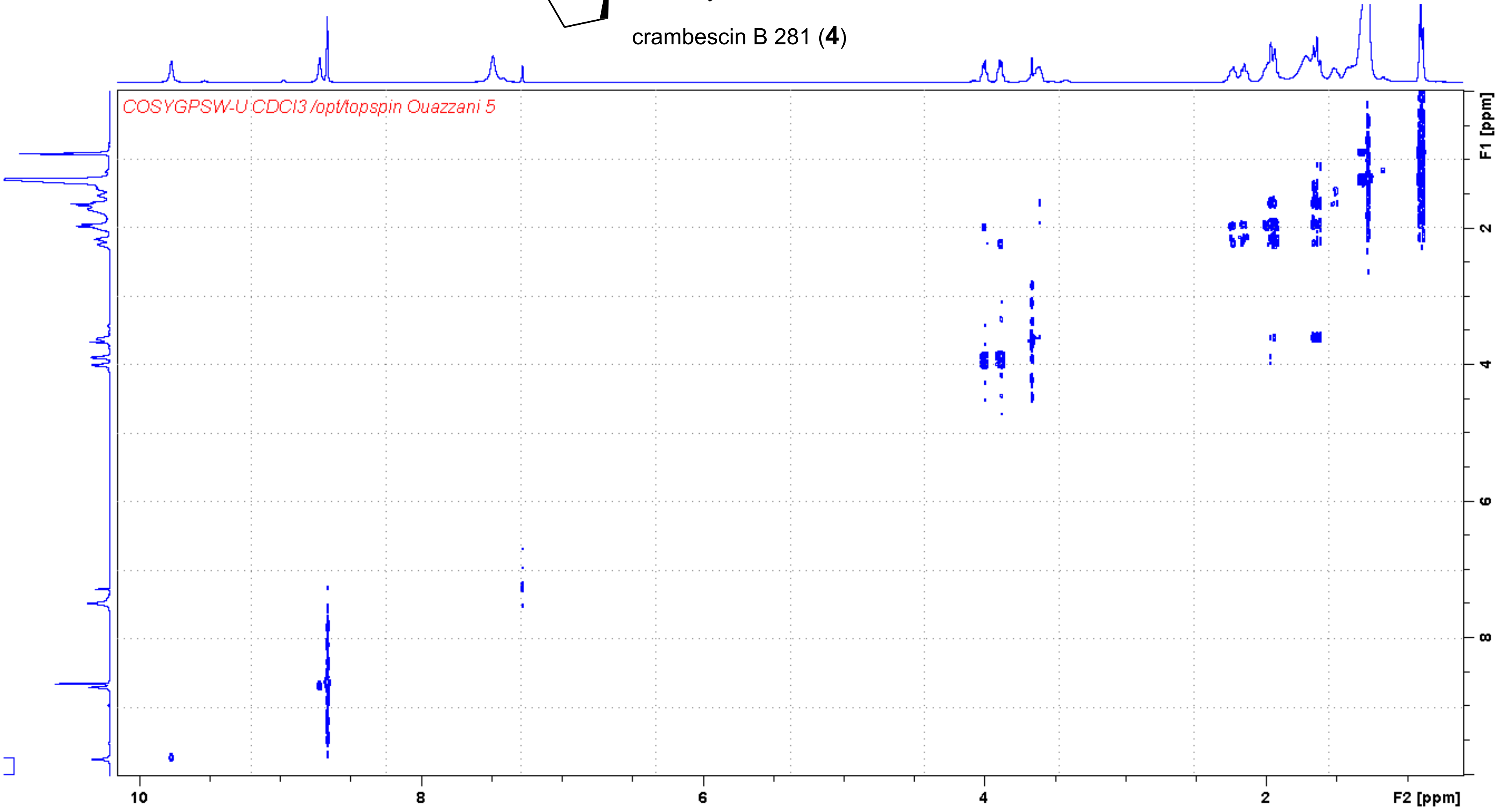

S19- crambescin B 281 (**4**) ^1H-^1H COSY spectrum (500 MHz, $CDCl_3$)

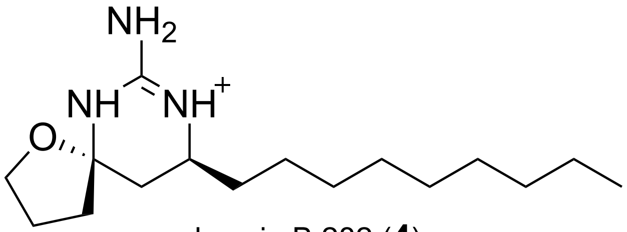

crambescin B 282 (**4**)

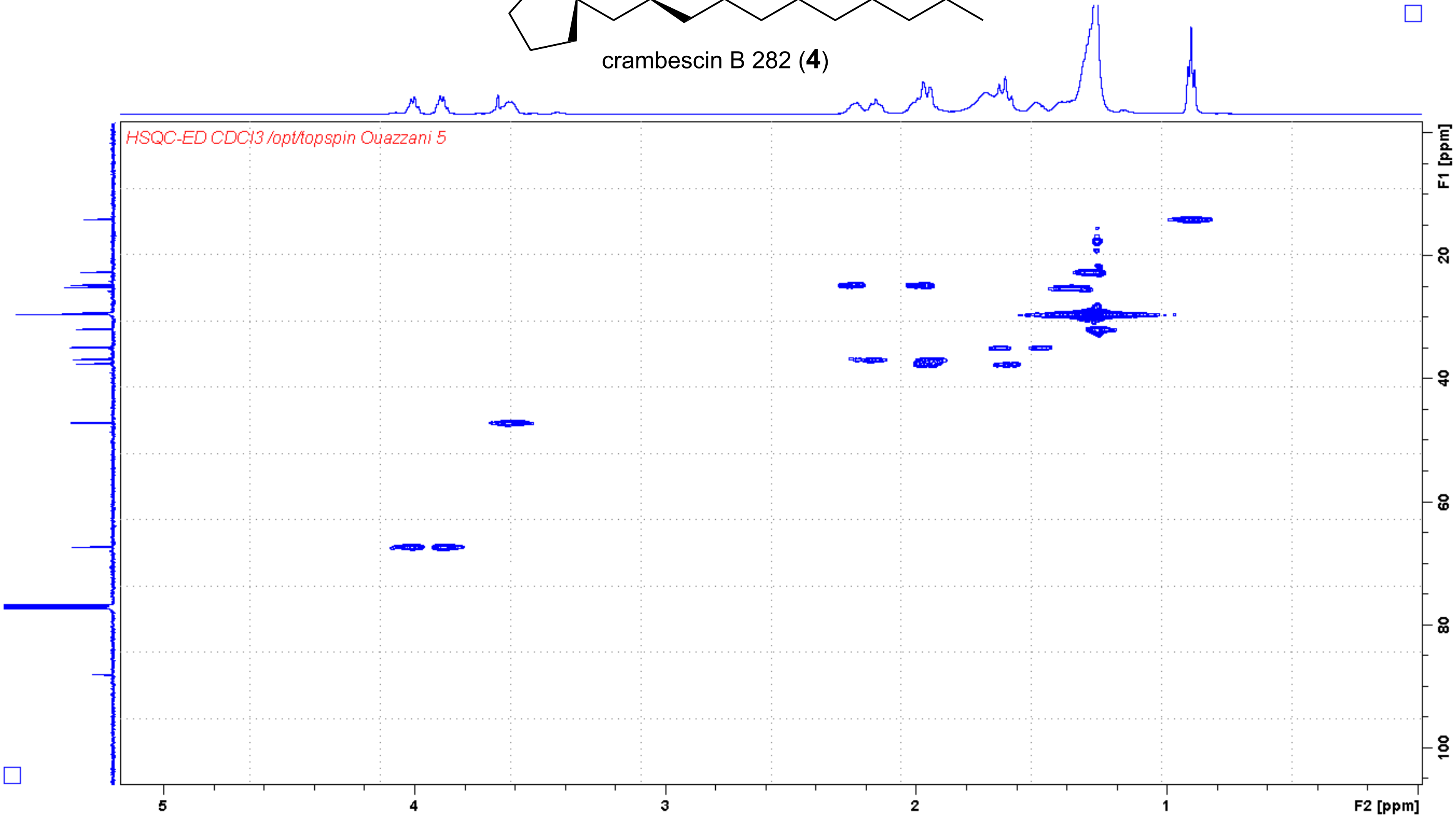

S20- crambescin B 282 (**4**) ^{1}H-^{13}C HSQC spectrum (500 MHz, $CDCl_3$)

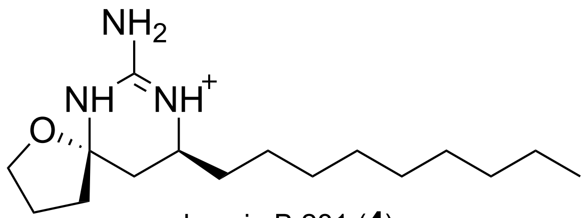

crambescin B 281 (**4**)

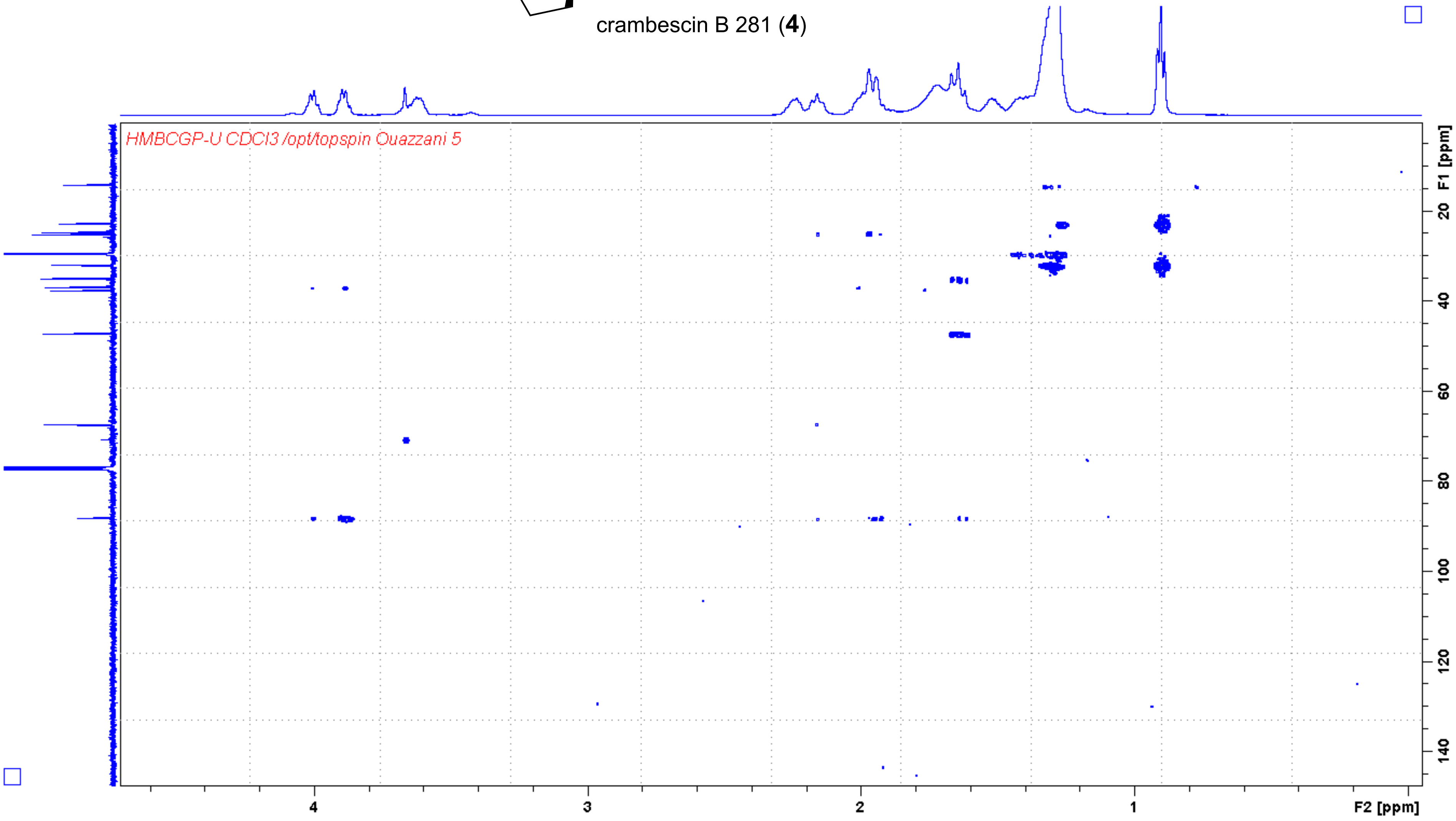

S21- crambescin B 281 (**4**) ^{1}H-^{13}C HMBC spectrum (500 MHz, $CDCl_3$)

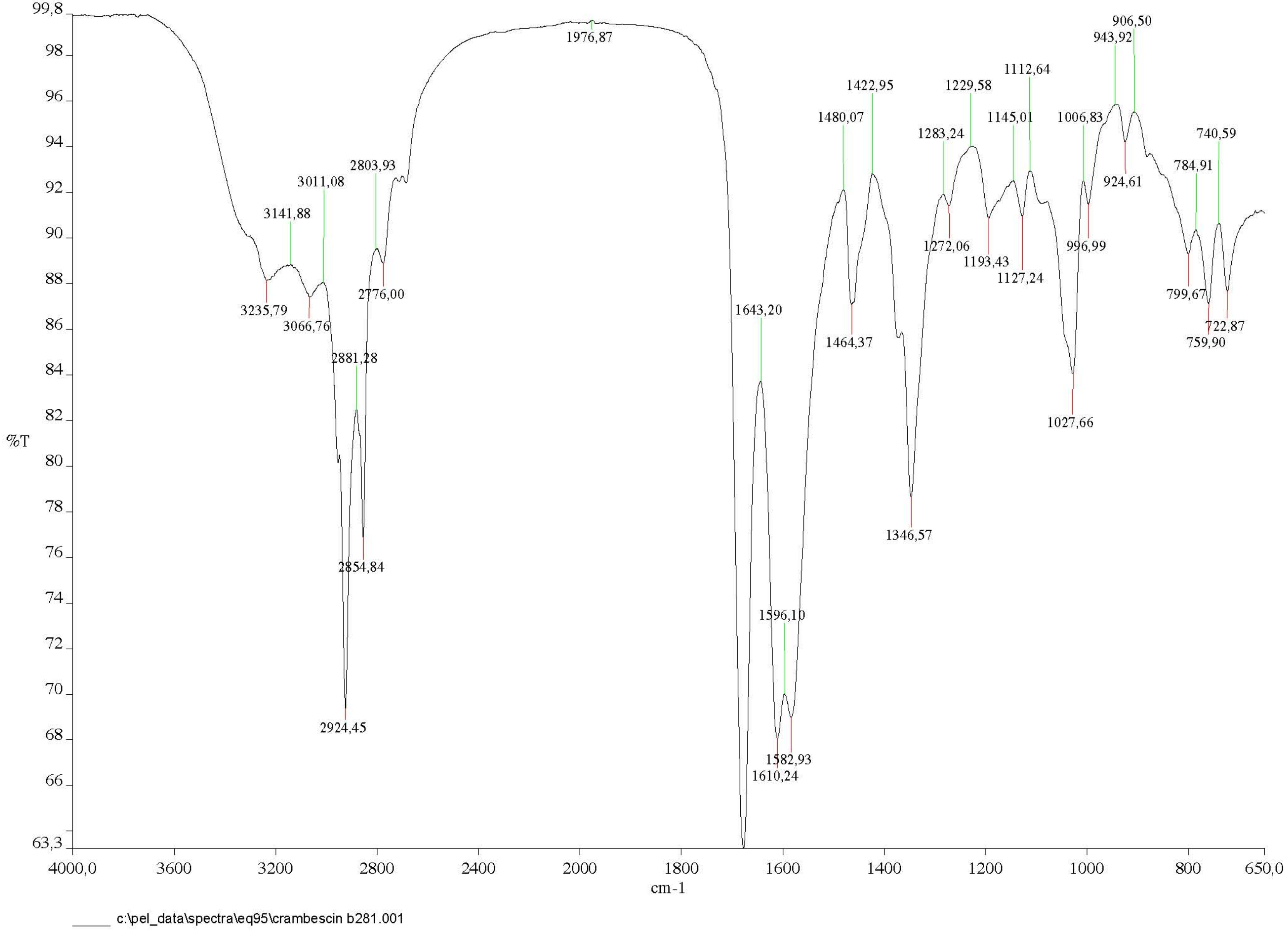

S22- crambescin B 281 (**4**) IR spectrum

Elemental Composition Report

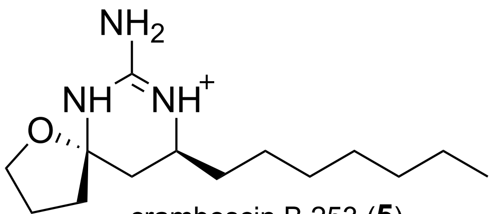

crambescin B 253 (**5**)

Single Mass Analysis

Tolerance = 20.0 PPM / DBE: min = -1.5, max = 100.0
Element prediction: Off
Number of isotope peaks used for i-FIT = 9

Monoisotopic Mass, Even Electron Ions
296 formula(e) evaluated with 2 results within limits (all results (up to 1000) for each mass)
Elements Used:
C: 0-50 H: 0-100 N: 0-10 O: 0-10

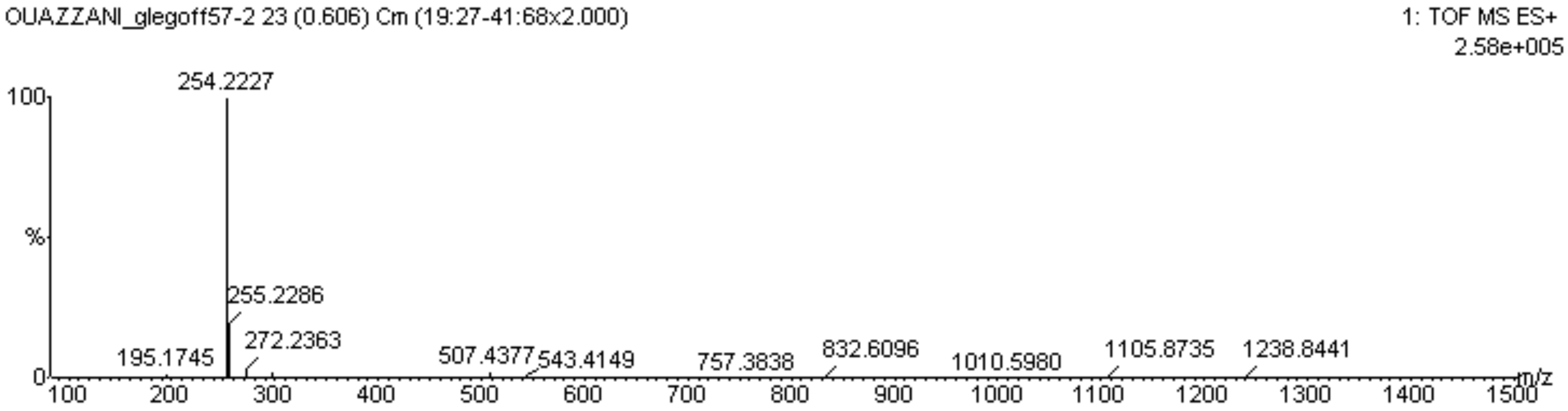

Minimum:				-1.5			
Maximum:		5.0	20.0	100.0			

Mass	Calc. Mass	mDa	PPM	DBE	i-FIT	i-FIT (Norm)	Formula
254.2227	254.2232	-0.5	-2.0	2.5	985.5	0.0	C14 H28 N3 O
	254.2192	3.5	13.8	-1.5	999.5	14.0	C9 H28 N5 O3

S23- crambescin B 253 (**5**) HRESIMS

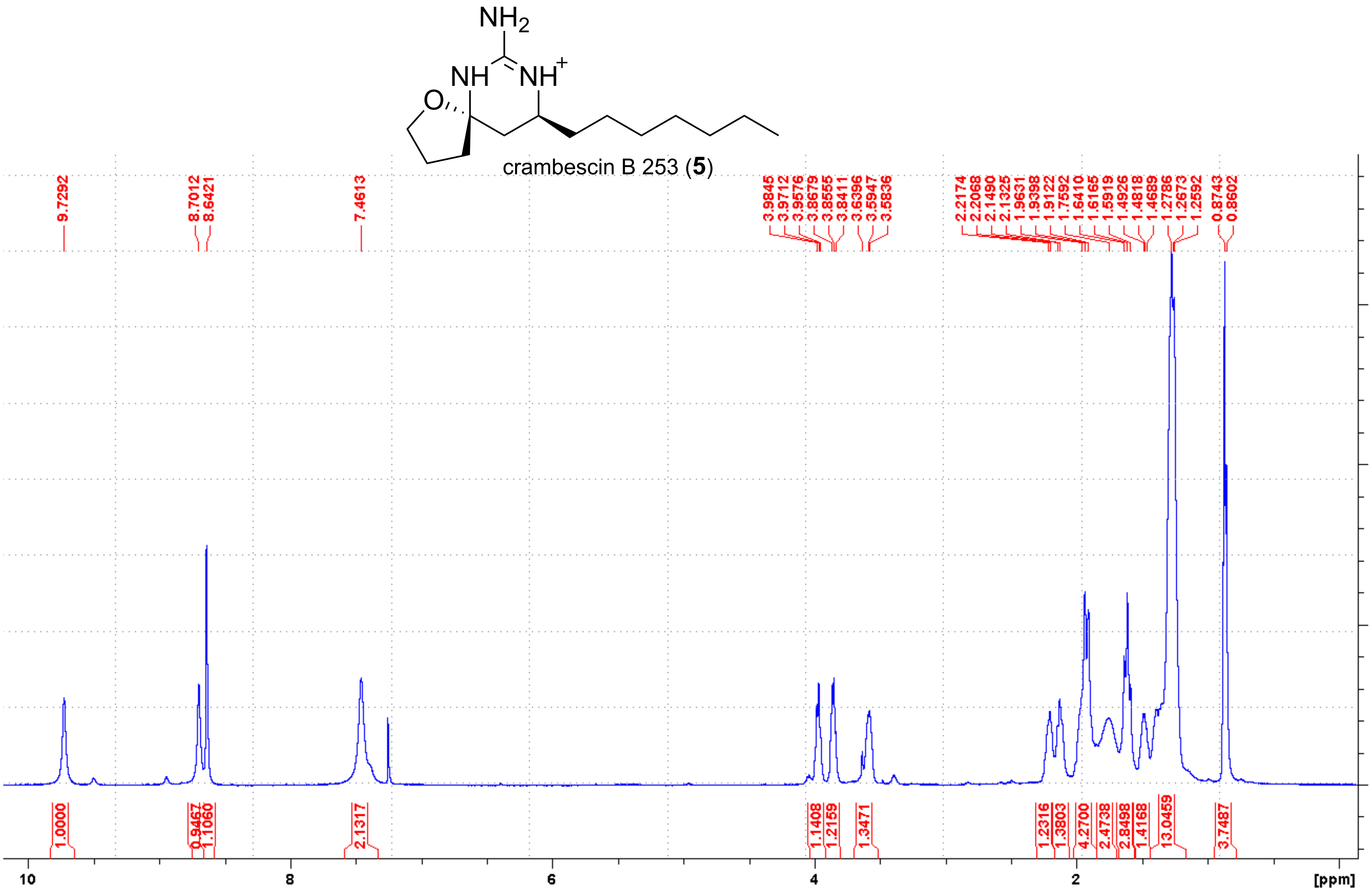

S24- crambescin B 253 (**5**) ^{1}H-NMR spectrum (500 MHz, $CDCl_3$)

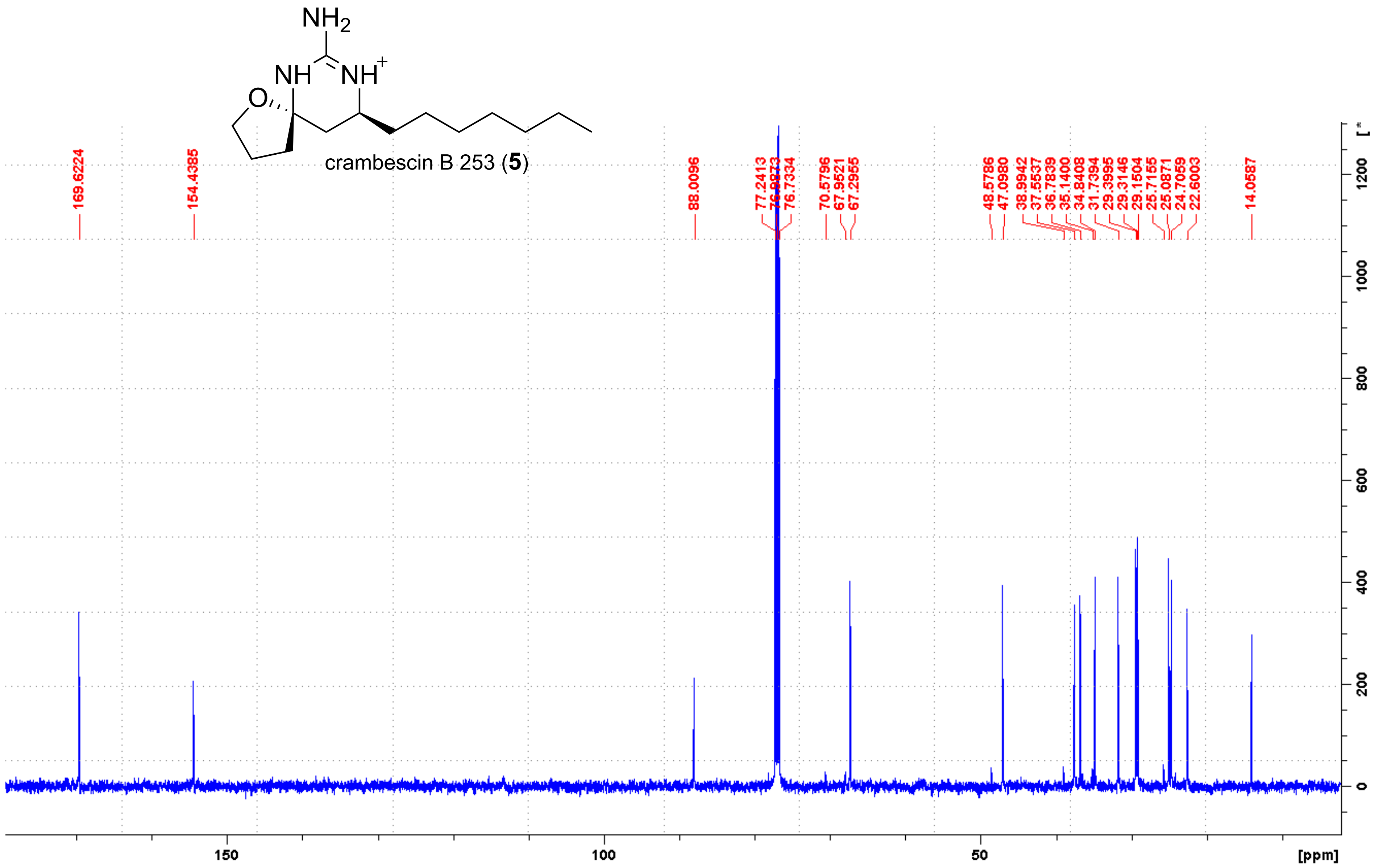

S25- crambescin B 253 (**5**) ^{13}C-NMR spectrum (125 MHz, $CDCl_3$)

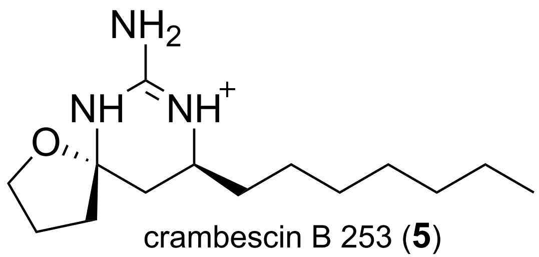

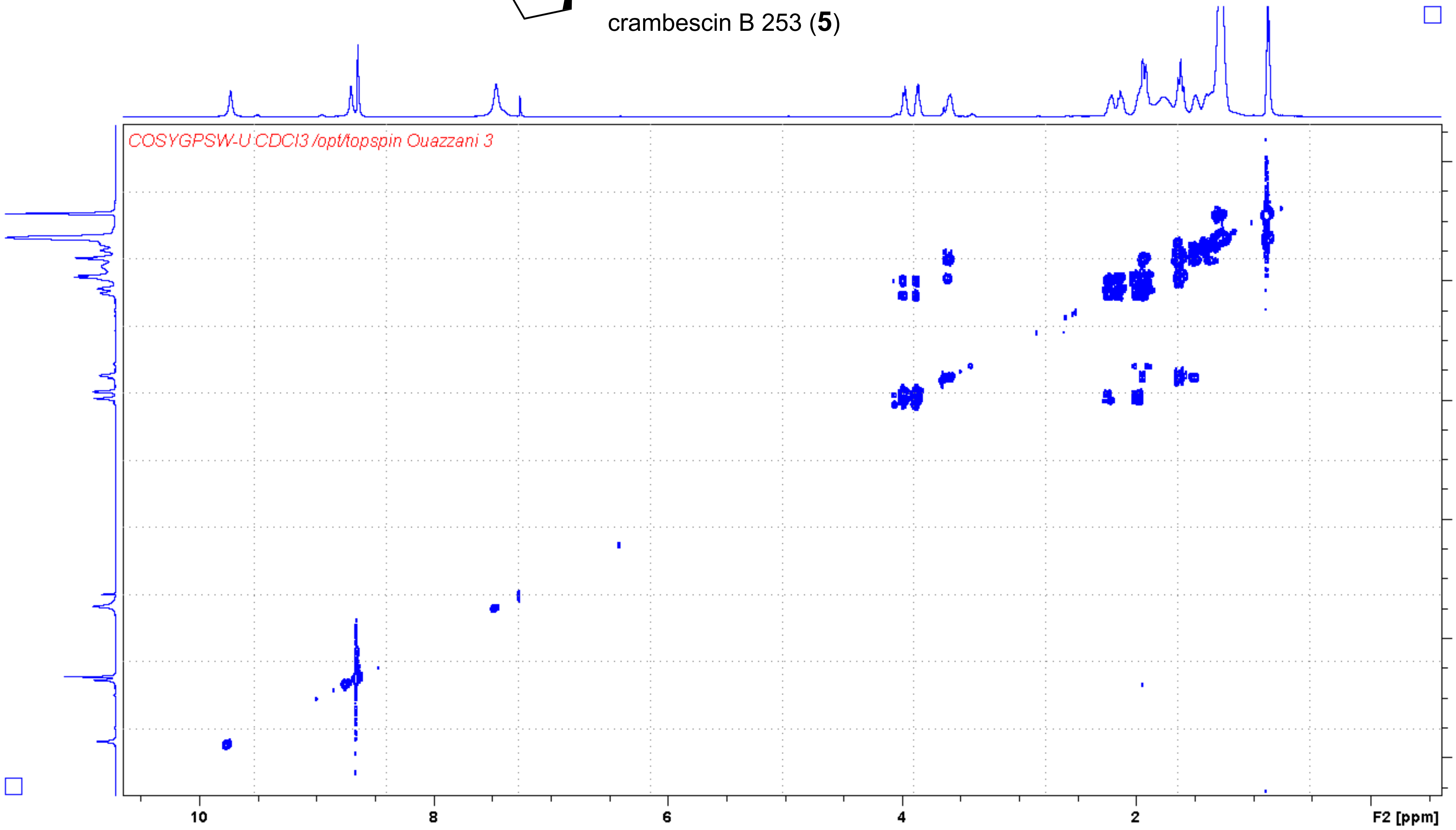

S26- crambescin B 253 (**5**) ^1H-^1H COSY spectrum (500 MHz, $CDCl_3$)

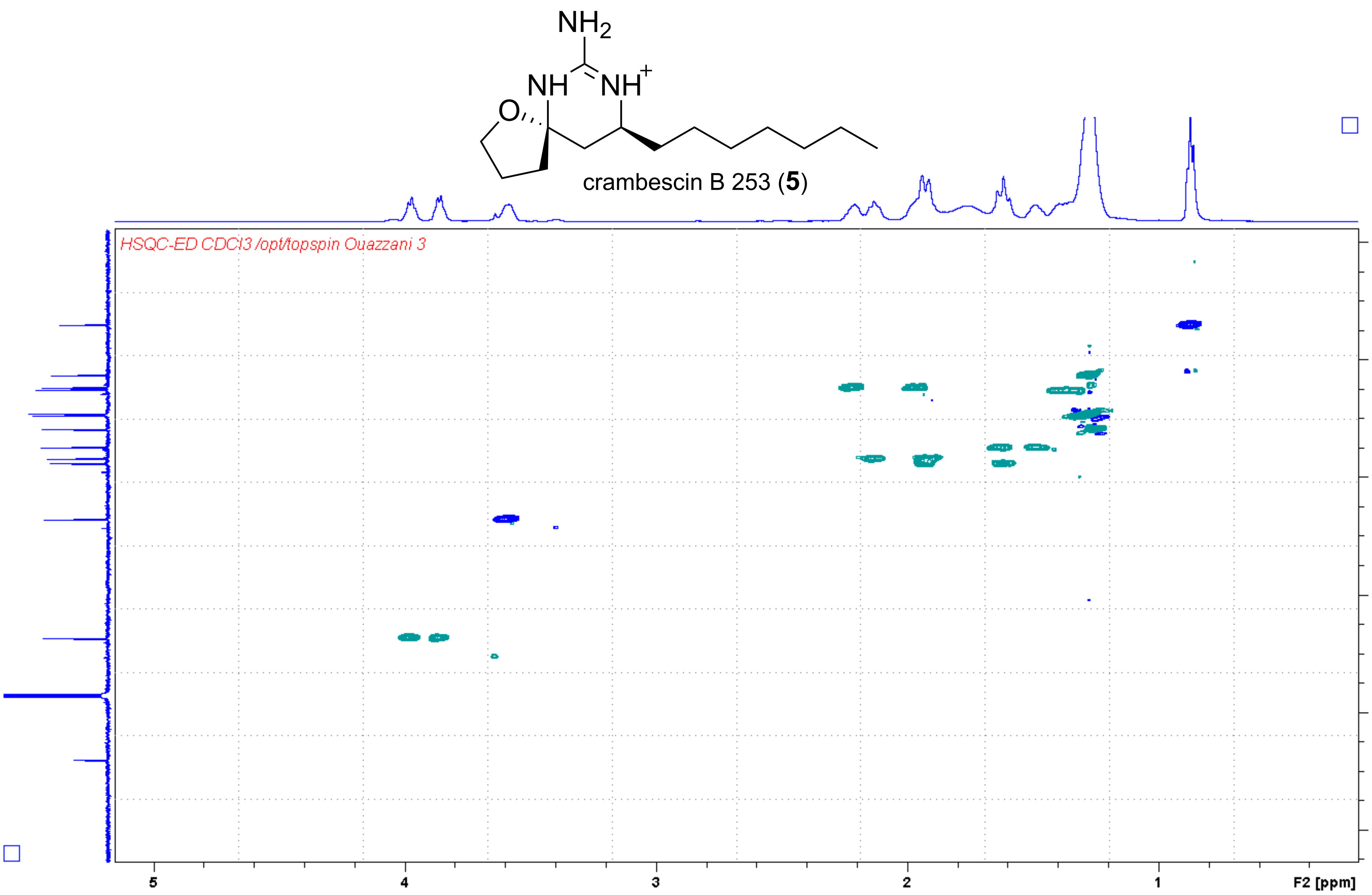

S27- crambescin B 253 (**5**) ^{1}H-^{13}C HSQC-ED spectrum (500 MHz, $CDCl_3$)

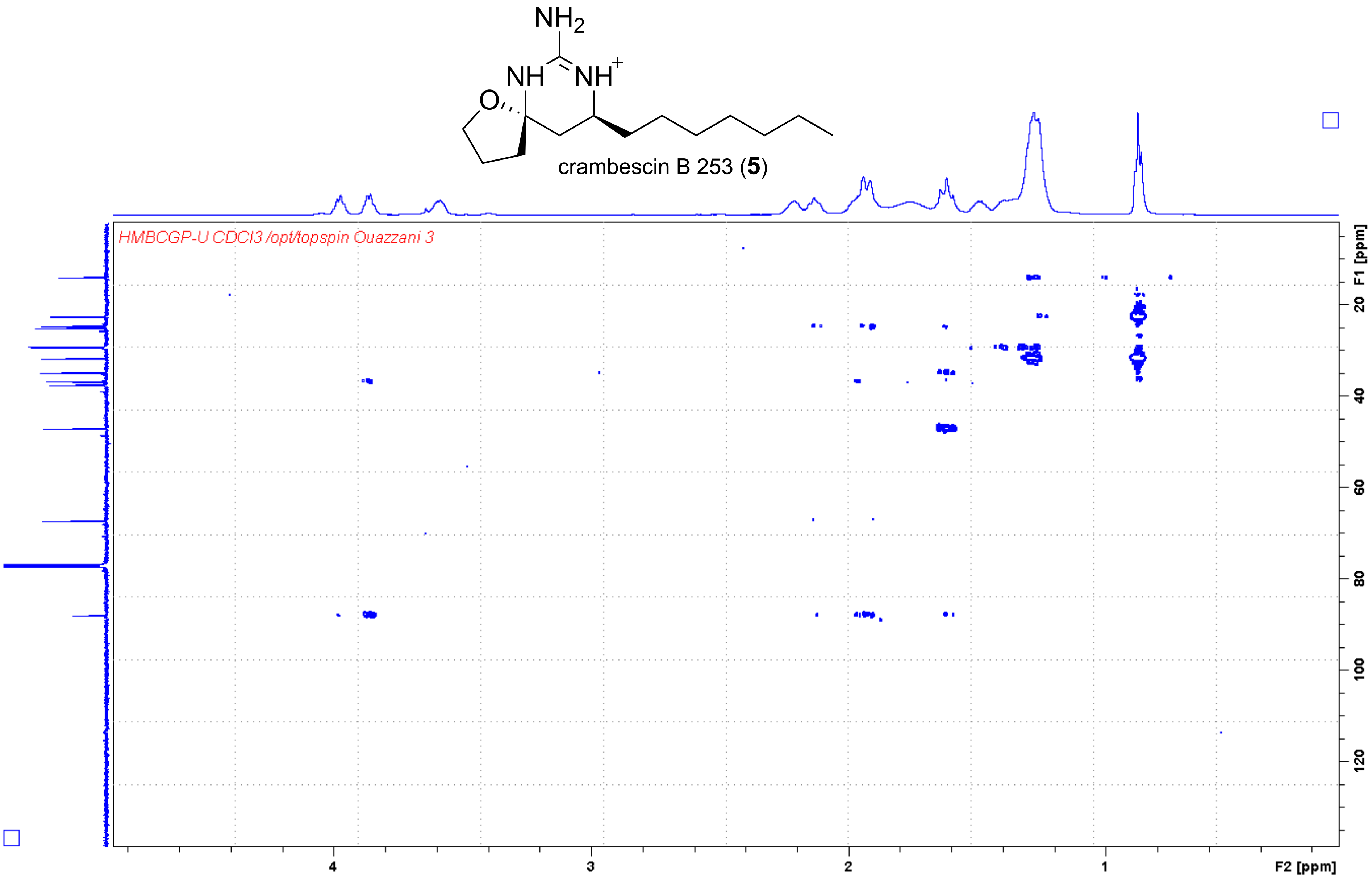

S28- crambescin B 253 (**5**) ^{1}H-^{13}C HMBC spectrum (500 MHz, $CDCl_3$)

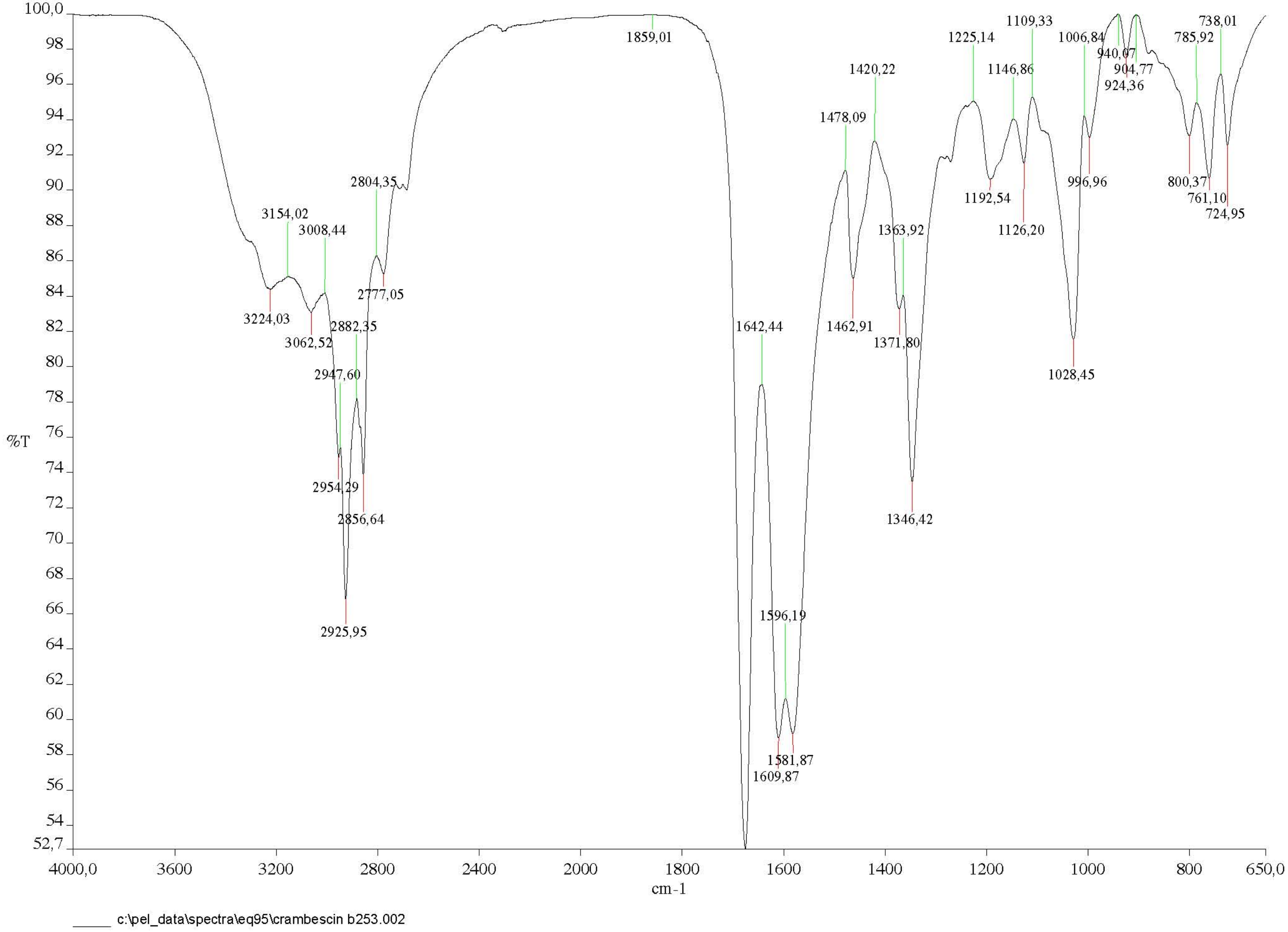

S29- crambescidin 8 253 (**5**) IR spectrum

	Crambescidin 359			Crambescidin acid			Crambescidin 401		
position	δ_C, type	δ_H,mult, *J* in Hz	HMBC	δ_C, type	δ_H,mult, *J* in Hz	HMBC	δ_C, type	δ_H,mult, *J* in Hz	HMBC
1	10.9, CH_3	0.84; t (*7.4*)	C-2, C-3	10.7, CH_3	0.80; t (*7.3*)	C-2, C-3	10.9, CH_3	0.88; m	C-2, C-3
2	30.3, CH_2	1.54, m; 1.46, m	-	30.8, CH_2	1.54, m; 1.47, m	-	30.2, CH_2	1.54, m; 1.46, m	-
3	72.2, CH	4.36, br d (*9.5*)	-	72.2, CH	4.34, br m	-	72.2, CH	4.56, br m	-
4	134.3, CH	5.51, br d (*11.1*)	C-3, C-6	134.5, CH	5.51, br d (*11.2*)	C-2, C-3, C-6	134.3, CH	5.54, br d (*11.1*)	C-3, C-6
5	131.4, CH	5.72, br dd (9.8, 11.1)	C-3	131.7, CH	5.72, br m	C-3, C-6, C-7	131.4, CH	5.76, br m	C-3, C-6, C-7
6	24.5, CH_2	2.15, m; 2.43, m	C-4, C-5, C-8	24.6, CH_2	2.15, m; 2.43, m	C-4, C-5, C-7, C-8	24.5, CH_2	2.15, m; 2.43, m	C-4, C-5, C-7, C-8
7	38.4, CH_2	1.97, dd (*5.9, 14.2*); 2.34, m	C-5, C-6, C-8, C-9	37.4, CH_2	2.02, m; 2.24 m	C-5, C-6, C-8, C-9	37.8, CH_2	2.02, m; 2.61 br t (*13.4*)	C-5, C-6, C-8, C-9
8	85.1, C	-	-	85.2, C	-	-	86.6, C	-	-
9	37.9, CH_2	1.47,m; 2.60 dd (*4.6, 13.1*)	C-8, C-10, C-11	37.8, CH_2	1.50 m; 2.53 dd (*3.3, 12.9*)	C-8, C-10, C-11	35.4, CH_2	1.6 t (*12.9*); 2.81 dd (*3.3, 12.9*)	C-8, C-10, C-11
10	55.1, CH	3.99-4.08, m	-	55.8, CH	4.00, m	C-9	60.1, CH	4.82, m	-
11	30.9, CH_2	1.74, m; 2.32, m	-	30.9, CH_2	1.74, m; 2.32, m	-	30.5, CH_2	2.06, m; 2.68, m	C-12, C-13
12	30.2, CH_2	1.46-1.54, br m	-	28.5, CH_2	2.06, m; 2.25, m	C-10, C-11,C-13	31.4, CH_2	3.38, m	C-10, C-11,C-13
13	53.5, CH	3.99-4.08, m	-	54.9, CH	4.21, m	C-12, C-14, C-22	160.7, C	-	-
14	40.4, CH_2	1.59, m; 2.60, dd (*4.7, 13.2*)	C-13, C-15, C-16	54.1, CH	2.68, d (*4.7*)	C-13, C-15, C-16, C-22	123.6, C	-	-
15	81.6, C	-	-	82.8, C	-	-	86.6, C	-	-
16	34.4, CH_2	1.69-1.75,br m	-	32.8, CH_2	1.27, m; 1.68, m	-	37.4, CH_2	3.01-3.08, br m	C-14,C-17, C-18, C-22
17	19.5, CH_2	1.82, m	-	19.8, CH_2	1.80, m; 1.87, m	C-15, C-16, C-22	25.1, CH_2	1.80, m; 1.87, m	C-15, C-16, C-22
18	33.3, CH_2	1.26, m; 1.69, m	C-17, C-19	34.5, CH_2	1.48, br m	-	39.5, CH_2	1.48, br m	-
19	66.3, CH	3.75, m	-	67.8, CH	3.81, m	C-17, C-18	68.0, CH	3.74, m	C-17
20	21.9, CH_3	1.08, d (*6.2*)	C-18, C-19	22.1, CH_3	1.08, d (*6.2*)	C-18, C-19	21.9, CH_3	1.14, d (*6.2*)	C-18, C-19
21	148.9, C	-	-	150.8, C	-	-	151.3, C	-	-
22	175.1, C	-	-	175.1, C	-	-	179.5, C	-	-

S30- ^{1}H NMR (500 MHz) and ^{13}C NMR (125 MHz) for crambescidin 359
^{1}H NMR (600 MHz cryoprobe) and ^{13}C NMR (150 MHz) for crambescidin acid and crambescidin 401

	Crambescin B 281			Crambescin B 253		
position	δ_C, type	δ_H,mult, *J* in Hz	HMBC	δ_C, type	δ_H,mult, *J* in Hz	HMBC
1	154.4, C	-	-	154.4, C	-	-
2	88.0, C	-	-	88.0, C	-	-
3	67.3, CH_2	3.86, 3.98; dd (*6.5*, *12.8*)	C-2, C-4	67.3, CH_2	3.86, 3.98; dd (*6.5*, *12.8*)	C-2, C-4
4	54.7, CH_2	1.97, 2.22; m	-	54.7, CH_2	1.97, 2.22; m	-
5	36.8, CH_2	1.91, 2.14; m	C-2, C-4	36.8, CH_2	1.91, 2.14; m	C-2, C-4
6	37.7, CH_2	1.62, 1.93; m	C-2, C-7, C-8, C-9	37.7, CH_2	1.62, 1.93; m	C-2, C-7, C-8, C-9
7	47.1, CH	3.59; m	-	47.1, CH	3.59; m	-
8	34.9, CH_2	1.49, 1.63; m	C-7	34.9, CH_2	1.49, 1.63; m	C-7
9	25.1, CH_2	1.22-1.14; m	-	25.1, CH_2	1.22-1.13; m	-
10	29.5, CH_2	1.22-1.14; m		29.4, CH_2	1.22-1.13; m	-
11	29.5, CH_2	1.22-1.14; m		29.2, CH_2	1.22-1.13; m	-
12	29.4, CH_2	1.22-1.14; m	-	38.1, CH_2	1.22-1.13; m	-
13	29.3, CH_2	1.22-1.14; m	-	22.9, CH_2	1.22-1.13; m	-
14	38.1, CH_2	1.22-1.14; m	-	14.1, CH_3	0.87; t (*7.1*)	C-12, C-13
15	22.9, CH_2	1.22-1.14; m	-			
16	14.1, CH_3	0.87; t (*7.1*)	C-14, C-15			

S31- ^{1}H NMR (500 MHz) and ^{13}C NMR (125 MHz) for crambescin 281 and crambescin 253
